# Iripin-1, a new anti-inflammatory tick serpin, inhibits leukocyte recruitment *in vivo* while altering the levels of chemokines and adhesion molecules

**DOI:** 10.3389/fimmu.2023.1116324

**Published:** 2023-01-23

**Authors:** Adéla Chlastáková, Barbora Kaščáková, Jan Kotál, Helena Langhansová, Michail Kotsyfakis, Ivana Kutá Smatanová, Lucas Tirloni, Jindřich Chmelař

**Affiliations:** ^1^ Department of Medical Biology, Faculty of Science, University of South Bohemia in České Budějovice, České Budějovice, Czechia; ^2^ Laboratory of Molecular Biology of Ticks, Institute of Parasitology, Biology Centre of the Czech Academy of Sciences, České Budějovice, Czechia; ^3^ Laboratory of Structural Chemistry, Institute of Chemistry, Faculty of Science, University of South Bohemia in České Budějovice, České Budějovice, Czechia; ^4^ Tick-Pathogen Transmission Unit, Laboratory of Bacteriology, Rocky Mountain Laboratories, National Institute of Allergy and Infectious Diseases, Hamilton, MT, United States; ^5^ Laboratory of Genomics and Proteomics of Disease Vectors, Institute of Parasitology, Biology Centre of the Czech Academy of Sciences, České Budějovice, Czechia

**Keywords:** serpin, iripin, ticks, ixodes ricinus, tick saliva, tick-host interaction, anti-inflammatory protein, cell migration

## Abstract

Serpins are widely distributed and functionally diverse inhibitors of serine proteases. Ticks secrete serpins with anti-coagulation, anti-inflammatory, and immunomodulatory activities *via* their saliva into the feeding cavity to modulate host’s hemostatic and immune reaction initiated by the insertion of tick’s mouthparts into skin. The suppression of the host’s immune response not only allows ticks to feed on a host for several days but also creates favorable conditions for the transmission of tick-borne pathogens. Herein we present the functional and structural characterization of Iripin-1 (*Ixodes ricinus* serpin-1), whose expression was detected in the salivary glands of the tick *Ixodes ricinus*, a European vector of tick-borne encephalitis and Lyme disease. Of 16 selected serine proteases, Iripin-1 inhibited primarily trypsin and further exhibited weaker inhibitory activity against kallikrein, matriptase, and plasmin. In the mouse model of acute peritonitis, Iripin-1 enhanced the production of the anti-inflammatory cytokine IL-10 and chemokines involved in neutrophil and monocyte recruitment, including MCP-1/CCL2, a potent histamine-releasing factor. Despite increased chemokine levels, the migration of neutrophils and monocytes to inflamed peritoneal cavities was significantly attenuated following Iripin-1 administration. Based on the results of *in vitro* experiments, immune cell recruitment might be inhibited due to Iripin-1-mediated reduction of the expression of chemokine receptors in neutrophils and adhesion molecules in endothelial cells. Decreased activity of serine proteases in the presence of Iripin-1 could further impede cell migration to the site of inflammation. Finally, we determined the tertiary structure of native Iripin-1 at 2.10 Å resolution by employing the X-ray crystallography technique. In conclusion, our data indicate that Iripin-1 facilitates *I. ricinus* feeding by attenuating the host’s inflammatory response at the tick attachment site.

## 1 Introduction

Serpins (serine protease inhibitors) are a large family of proteins that are present in all three superkingdoms of life (Archaea, Bacteria, and Eukarya) as well as in viruses (1, 2). Owing to their ability to inhibit serine proteases, serpins are involved in the regulation of many important physiological processes, including blood coagulation, the complement cascade, fibrinolysis, angiogenesis, inflammation, and tissue remodeling (3). Serpins are typically composed of 350–400 amino acids and have an average molecular weight between 40–60 kDa (4). Despite low sequence homology, serpins share a highly conserved tertiary structure that usually consists of three β-sheets (A, B, C), eight to nine α-helices, and a reactive center loop (RCL) (5, 6). Serpins inhibit proteases through a unique suicide substrate-like mechanism, which results in the inactivation of not only the protease but also the serpin (7). Initially, recognition of the P1 site of the serpin RCL by the protease leads to the creation of a non-covalent Michaelis-Menten-like complex. The bound protease subsequently cleaves the scissile bond between the P1 and P1’ residues of the RCL, resulting in covalent bond formation between the protease and the serpin. Finally, the cleaved RCL inserts into the center of the β-sheet A to form an extra strand, and the covalently bound protease is translocated to the opposite end of the serpin molecule (4, 7, 8).

*Ixodes ricinus* (Acari: Ixodidae) is a European tick species that transmits several animal and human pathogens, including tick-borne encephalitis virus and *Borrelia burgdorferi* sensu lato (9). It is a highly specialized obligate ectoparasite whose three active developmental stages (larva, nymph, adult female) must blood feed on different hosts in order to molt or lay eggs (10). The insertion of the tick hypostome and two chelicerae into skin triggers the host’s hemostatic and inflammatory response (10). In reaction to skin integrity disruption and presence of foreign antigens, resident cells in the vicinity of the tick attachment site, such as keratinocytes, fibroblasts, mast cells, macrophages, and dendritic cells, start producing pro-inflammatory cytokines and chemokines (11). Pro-inflammatory cytokines tumor necrosis factor (TNF) and interleukin (IL)-1β stimulate the expression of cell adhesion molecules on the surface of endothelial cells (12). Chemokines made by resident cells assist in the firm adhesion of leukocytes to endothelial cells and also create a concentration gradient in the extravascular space which directs innate immune cells (such as neutrophils and monocytes) to the tick feeding site (13).

Leukocyte extravasation from blood into injured tissues involves a series of sequential and overlapping steps. Leukocytes, rolling along the inflamed endothelium, bind through their G protein-coupled receptors to chemokines attached to the luminal side of the blood vessel wall (13). The interaction between chemokines and their receptors triggers a conformation change in leukocyte integrins that allows tight binding of integrins to their ligands on endothelial cells (14). The integrins lymphocyte function-associated antigen-1 (LFA-1, CD11a/CD18) and macrophage-1 antigen (Mac-1, CD11b/CD18) bind to intercellular adhesion molecule-1 (ICAM-1), and the integrin very late antigen-4 (VLA-4, CD49d/CD29) interacts with vascular cell adhesion molecule-1 (VCAM-1) (15). Binding of integrins to their ligands enables the firm adhesion of leukocytes to endothelium (14). Firm adhesion and crawling of leukocytes are followed by transendothelial migration that involves homophilic as well as heterophilic interactions between molecules expressed on both leukocytes and endothelial cells, such as platelet/endothelial cell adhesion molecule-1 (PECAM-1), CD99, and junctional adhesion molecule-1 and -3 (JAM-1 and JAM-3) (16, 17). After crossing the pericyte layer and the vascular basement membrane, leukocytes migrate through the extravascular space to the site of tissue injury (18). Over the course of migration, cells attach *via* integrins to extracellular matrix (ECM) components, such as collagen, laminin, and fibronectin, and simultaneously engage membrane-associated and secreted proteases to remodel and penetrate the ECM barrier (15, 19).

Ticks must suppress inflammation accompanied by itch and pain so that they could feed uninterruptedly on a host for several days (10). For this reason, ticks secrete saliva, containing anti-inflammatory and immunomodulatory molecules, into the wound (20, 21). Based on their presence in tick saliva and the ability to modulate immune reaction, serpins are one of the protein families that ticks utilize to attenuate the host’s inflammatory response (22). To date, tick serpins have been shown to reduce vascular permeability (23–25), suppress the production of pro-inflammatory cytokines and chemokines (26–30), and attenuate nitric oxide production by macrophages (31). Moreover, *I. ricinus* salivary serpins Iripin-5 and IRS-2 inhibited neutrophil migration in the transwell assay and in the mouse model of paw edema, respectively. However, the reasons behind the impaired migration of neutrophils in the presence of both serpins remain unclear (31, 32).

In this study, we tested the effect of *I. ricinus* salivary serpin Iripin-1 on innate immune responses by utilizing a combination of *in vitro* assays and the *in vivo* thioglycolate-induced peritonitis model. Iripin-1 did not significantly affect the production of pro-inflammatory cytokines but increased the secretion of the anti-inflammatory cytokine IL-10. In addition to IL-10, Iripin-1 enhanced the production of chemokines involved in the recruitment of neutrophils (KC/CXCL1, MIP-2/CXCL2), monocytes (MCP-1/CCL2), and eosinophils (eotaxin-1/CCL11). Despite increased chemokine levels, Iripin-1 inhibited the recruitment of neutrophils and monocytes, but not eosinophils, to the site of inflammation. The inhibition of neutrophil and monocyte migration might be a result of the Iripin-1 ability to inhibit serine proteases (namely trypsin, kallikrein, matriptase, and plasmin) facilitating immune cell migration. Moreover, Iripin-1 decreased the expression of molecules engaged in leukocyte extravasation on the surfaces of both endothelial cells (VCAM-1, CD99) and neutrophils (chemokine receptor CXCR2), which might also contribute to the observed inhibition of immune cell recruitment. Based on the aforementioned experimental results, we conclude that *I. ricinus* ticks utilize Iripin-1 to attenuate the inflammatory response initiated by the insertion of tick mouthparts into host’s skin. Besides determining the function, we also revealed the tertiary structure of native Iripin-1 by employing X-ray crystallography.

## 2 Materials and methods

### 2.1 Animals

Pathogen-free *I. ricinus* ticks were obtained from the tick colony maintained at the Institute of Parasitology, Biology Centre of the Czech Academy of Sciences (IP BC CAS), České Budějovice, Czechia. Guinea pigs utilized for *I. ricinus* feeding were also bred and maintained at IP BC CAS. C57BL/6N mice were purchased from Velaz (Prague, Czechia) and subsequently were housed in individually ventilated cages in the animal house facility of the Department of Medical Biology, Faculty of Science, University of South Bohemia in České Budějovice, Czechia. The mice were maintained in a 12 h light/12 h dark cycle and were provided with a standard pellet diet and water *ad libitum*. Mice at the age of 8–16 weeks were utilized in all immunological experiments. Animal experiments were conducted in accordance with the Animal Protection Law of the Czech Republic No. 246/1992 Sb. and the protocols approved by the Czech Academy of Sciences (experimental project No. 098/2020) and the Ministry of Education, Youth and Sports of the Czech Republic (experimental project No. 19085/2015-3).

### 2.2 *Iripin-1* expression in adult *I. ricinus* ticks

*I. ricinus* adult females were fed on guinea pigs for 1, 2, 3, 4, 6, and 8 days. Salivary glands, midguts, and ovaries were dissected from tick bodies under RNase-free conditions, and total RNA was extracted from tick tissues using TRI Reagent (Molecular Research Center, Cincinnati, OH, USA). Total RNA was reverse transcribed into cDNA using the Transcriptor First Strand cDNA Synthesis Kit (Roche Diagnostics, Mannheim, Germany) according to the manufacturer’s instructions. The prepared cDNA, mixed with FastStart Universal SYBR Green Master (Roche Diagnostics) and gene-specific primers, was subsequently utilized for the analysis of *iripin-1* transcription by RT-qPCR in the thermal cycler Rotor-Gene 6000 (Corbett Research, Cambridge, UK). Cycling conditions were 95°C for 10 min followed by 45 cycles of 95°C for 15 s, 60°C for 10 s, and 72°C for 30 s. The relative expression of *iripin-1* in the organs of adult females was calculated according to the 2^-ΔΔCt^ (Livak) method (33). The gene encoding ribosomal protein S4 (*rps4*, GenBank accession number MN728897.1) was utilized for the normalization of *iripin-1* expression. The nucleotide sequences of used primers and amplicon lengths are provided in **Supplementary Table 1**.

### 2.3 Production of recombinant Iripin-1

To determine the crystal structure and function of Iripin-1, recombinant protein was prepared in the *Escherichia coli* expression system, and endotoxin was removed by a detergent-based method. The whole process of Iripin-1 production is described in detail in the **Supplementary Material**. The endotoxin-free buffer (PBS, pH 7.4, 100 mM betaine, 1 mM EDTA, and 10% glycerol), in which recombinant Iripin-1 was dissolved, was used in all immunological experiments as a control.

### 2.4 Inhibition of serine proteases

Initially, the inhibitory specificity of Iripin-1 against selected serine proteases was evaluated by using the method described previously (32). Briefly, each protease (in the amount shown in **Supplementary Table 2**) and 200 nM Iripin-1 were incubated for 10 min at room temperature before addition of an appropriate fluorogenic substrate at 250 μM final concentration. The substrate hydrolysis rate in the presence and absence of Iripin-1 was determined with the help of the SpectraMax Gemini XPS Microplate Reader (Molecular Devices, San Jose, CA, USA). Formation of covalent complexes between Iripin-1 and proteases with enzymatic activity reduced by more than 50% in the initial screening experiment was assessed as follows. Iripin-1 and proteases were incubated at 1 μM final concentrations in 20 mM Tris, 150 mM NaCl, 0.01% Tween 20, pH 7.4 for 1 h at 37°C. Covalent complex formation was subsequently analyzed by a reducing SDS-PAGE using 4–20% Criterion TGX gels (Bio-Rad Laboratories, Hercules, CA, USA), followed by Coomassie staining. Finally, inhibition constants for the interaction between Iripin-1 and trypsin, kallikrein, matriptase, and plasmin were measured by a discontinuous method under pseudo-first-order conditions, as detailed in the **Supplementary Material**.

### 2.5 Production of cytokines and chemokines by resident peritoneal cells

Resident peritoneal cells (RPCs) were harvested by washing the peritoneal cavities of naïve C57BL/6 mice with 10 mL of ice-cold Hanks’ balanced salt solution (Biosera, Nuaillé, France). Following the removal of red blood cells by the addition of 1x RBC lysis buffer (Invitrogen, Waltham, MA, USA), RPCs were resuspended in RPMI 1640 medium with stable glutamine (Biosera) supplemented with 0.5% bovine serum albumin (BSA, Biosera) and seeded into a 24-well cell culture plate (3.5 × 10^5^ cells in 300 μL of culture medium per well). After incubating the cells for 4 h at 37°C and 5% CO_2_, the culture medium was replaced with fresh medium, and Iripin-1 (2 μM) or the corresponding amount of control buffer were added to certain wells. Following 30 min incubation at 37°C and 5% CO_2_, RPCs were stimulated by the addition of 1 μg/mL lipopolysaccharide (LPS, *E. coli* serotype O111:B4, Sigma-Aldrich, St. Louis, MO, USA) or were left unstimulated. Cell-free supernatants were collected after culturing the cells for 4 h at 37°C and 5% CO_2_ in the presence or absence of LPS and were stored at – 80°C until use. The concentrations of selected cytokines and chemokines in the supernatants were measured by commercial sandwich enzyme-linked immunosorbent assay (ELISA) kits as described later in the text.

### 2.6 Thioglycolate-induced peritonitis

C57BL/6 mice were randomly divided into two groups: a control group and an Iripin-1-treated group. Mice in the latter group were injected intraperitoneally (ip) with Iripin-1 (2 mg/kg) in saline (0.9% NaCl) solution and 30 min later, they were given another ip injection consisting of 200 μL 3% Difco thioglycolate medium (TGM, BD, Franklin Lakes, NJ, USA) and the second dose of Iripin-1 (2 mg/kg). Mice in the control group were treated identically to the mice in the Iripin-1 group except that control buffer was used instead of Iripin-1. Mice in both groups were sacrificed by cervical dislocation 4 h after TGM administration, and their peritoneal cavities were washed with 10 mL of ice-cold mixture of PBS and 5 mM EDTA to harvest recruited inflammatory cells. Following centrifugation, cell-free peritoneal lavage fluid was stored at – 80°C for later detection of cytokine and chemokine levels by a sandwich ELISA, and cell pellets were resuspended in FACS buffer (PBS, 0.1% BSA, 0.1% (w/v) sodium azide). Resuspended cells were counted using the Neubauer chamber and light microscope and subsequently were stained for flow cytometry. First of all, Fc receptors were blocked with anti-CD16/CD32 antibody (clone 93, Invitrogen), and then the following fluorophore-conjugated monoclonal antibodies were utilized to differentiate between neutrophils (CD11b^+^ Ly-6G^+^ Ly-6C^+^), monocytes (CD11b^+^ Ly-6C^high^), and eosinophils (Siglec-F^+^): anti-CD11b-FITC (clone M1/70) and anti-Ly-6C-APC (clone HK1.4) from Invitrogen, and anti-Ly-6G-PE (clone 1A8) and anti-Siglec-F-PerCP/Cyanine5.5 (clone E50-2440) from BD Biosciences. Finally, propidium iodide staining solution (Invitrogen) was applied to exclude nonviable cells. Flow cytometric analysis was performed on the BD FACSCanto II flow cytometer using BD FACSDiva software 6.1.3 (BD Biosciences). The images of dot plots were prepared in the program NovoExpress 1.4.1 (Agilent Technologies, Santa Clara, CA, USA).

### 2.7 ELISA

The levels of TNF, IL-1β, IL-6, IL-10, keratinocyte chemoattractant (KC/CXCL1), monocyte chemoattractant protein-1 (MCP-1/CCL2), regulated on activation, normal T cell expressed and secreted (RANTES/CCL5), and eotaxin-1/CCL11 were measured by Mouse DuoSet ELISA kits (R&D Systems, Minneapolis, MN, USA), and the level of macrophage inflammatory protein-2 (MIP-2/CXCL2) was determined using the Mouse MIP2 Matched Antibody Pair Kit (Abcam, Cambridge, UK). The ELISA kits from both manufacturers were used in combination with the DuoSet ELISA Ancillary Reagent Kit 2 (R&D Systems). All assays were performed according to the manufacturers’ instructions with only minor modifications, and optical densities at 450 and 540 nm were measured on the Synergy H1 microplate reader (BioTek Instruments, Winooski, VT, USA).

### 2.8 Expression of molecules on the surface of neutrophils

Bone marrow cells, isolated from femora and tibiae of C57BL/6 mice, were forced through a Falcon 40 μm cell strainer to obtain a single cell suspension, and contaminating erythrocytes were lysed by the addition of 1x RBC lysis buffer (Invitrogen). Neutrophils were obtained from the suspension of bone marrow cells by magnetic-activated cell sorting (negative selection) using the Neutrophil Isolation Kit (Miltenyi Biotec, Bergisch Gladbach, Germany) according to the instructions of the manufacturer. The purity of neutrophils isolated this way was approximately 85% (data not shown). Following their isolation, neutrophils were resuspended in RPMI 1640 medium with stable glutamine supplemented with 10% heat-inactivated fetal bovine serum, 100 U/mL penicillin G, 100 μg/mL streptomycin (all from Biosera), and 50 μM 2-mercaptoethanol (Sigma-Aldrich) and were seeded into a 96-well cell culture plate (1 × 10^5^ cells in 100 μL of culture medium per well). After seeding, neutrophils were incubated with Iripin-1 (2 μM) or the corresponding amount of control buffer for 30 min at 37°C and 5% CO_2_, and then were stimulated by the addition of 1 μg/mL LPS (*E. coli* serotype O111:B4, Sigma-Aldrich). After 3h incubation in the presence of buffer/Iripin-1 and LPS at 37°C and 5% CO_2_, neutrophils were collected from the culture plate and prepared for flow cytometric analysis. First, neutrophils in PBS were stained with the fixable viability dye eFluor 506 (Invitrogen) to exclude dead cells from the analysis. Afterwards, the cells were transferred from PBS to FACS buffer with added Super Bright Complete Staining Buffer (Invitrogen) and incubated with the following fluorophore-conjugated monoclonal antibodies: anti-Ly-6G-Super Bright 600 (clone 1A8-Ly6g), anti-CD11a-eFluor 450 (clone M17/4), anti-CD11b-Super Bright 780 (clone M1/70), anti-CD18-FITC (clone M18/2), and anti-CD31 (PECAM-1)-PE-Cyanine7 (clone 390) from Invitrogen and anti-CD182 (CXCR2)-PerCP/Cyanine5.5 (clone SA044G4) from BioLegend (San Diego, CA, USA). After washing the cells with FACS buffer, the data were collected and analyzed using the NovoCyte 3000 flow cytometer and software NovoExpress 1.4.1 (Agilent Technologies).

### 2.9 HUVEC culture and stimulation

At the beginning of each experiment, human umbilical vein endothelial cells (HUVECs), purchased from Lonza Group (Basel, Switzerland), were seeded into a 24-well cell culture plate coated with 0.5% gelatin in PBS. The seeding density was 1.2 × 10^5^ cells in 500 μL of complete culture medium per well. The complete growth medium used was endothelial basal medium EBM Plus supplemented with the components of the EGM Plus SingleQuot Kit (Lonza Group). After 1-day culture at 37°C and 5% CO_2_, the complete medium was replaced with starving medium of the following composition: EBM Plus basal medium + L-glutamine, gentamicin sulfate, and amphotericin B (all from the SingleQuot Kit) + 0.5% BSA (Biosera). Following overnight incubation, the medium was replaced with fresh starving medium, and the cells were pre-incubated with Iripin-1 (2 μM) or with the corresponding amount of control buffer for 30 min. Subsequently, 10 ng/mL TNF (Gibco, Thermo Fisher Scientific, Waltham, MA, USA) was added to certain wells, and the cells were cultured in the presence of buffer, Iripin-1, and TNF for 4 h or 6 h at 37°C and 5% CO_2_. HUVECs between passages 5 and 7 were used in all experiments. The RT-qPCR and flow cytometric analyses of the expression of cell adhesion molecules by HUVECs are described in the **Supplementary Material**.

### 2.10 Statistics

All graphs were prepared in the program GraphPad Prism 9.3.1 (GraphPad Software, San Diego, CA, USA). Differences between two groups were analyzed by unpaired two-tailed *t*-test. Differences between more than two groups were evaluated by one-way analysis of variance (ANOVA) followed by Dunnett’s *post hoc* test or by randomized block ANOVA, which involved two variables: a fixed effect factor (cell treatment) and a random effect factor/block (an experimental run) (34). The randomized block ANOVA was followed by Tukey’s *post hoc* test to find means, which were significantly different from each other. All statistical analyses were conducted in the program Statistica 13.5.0.17 (TIBCO Software, Palo Alto, CA, USA). Statistically significant differences between groups are marked with asterisks (* p < 0.05, ** p < 0.01, *** p < 0.001, **** p < 0.0001).

## 3 Results

### 3.1 *Iripin-1* expression is upregulated in the salivary glands of feeding ticks

**Figure 1:**
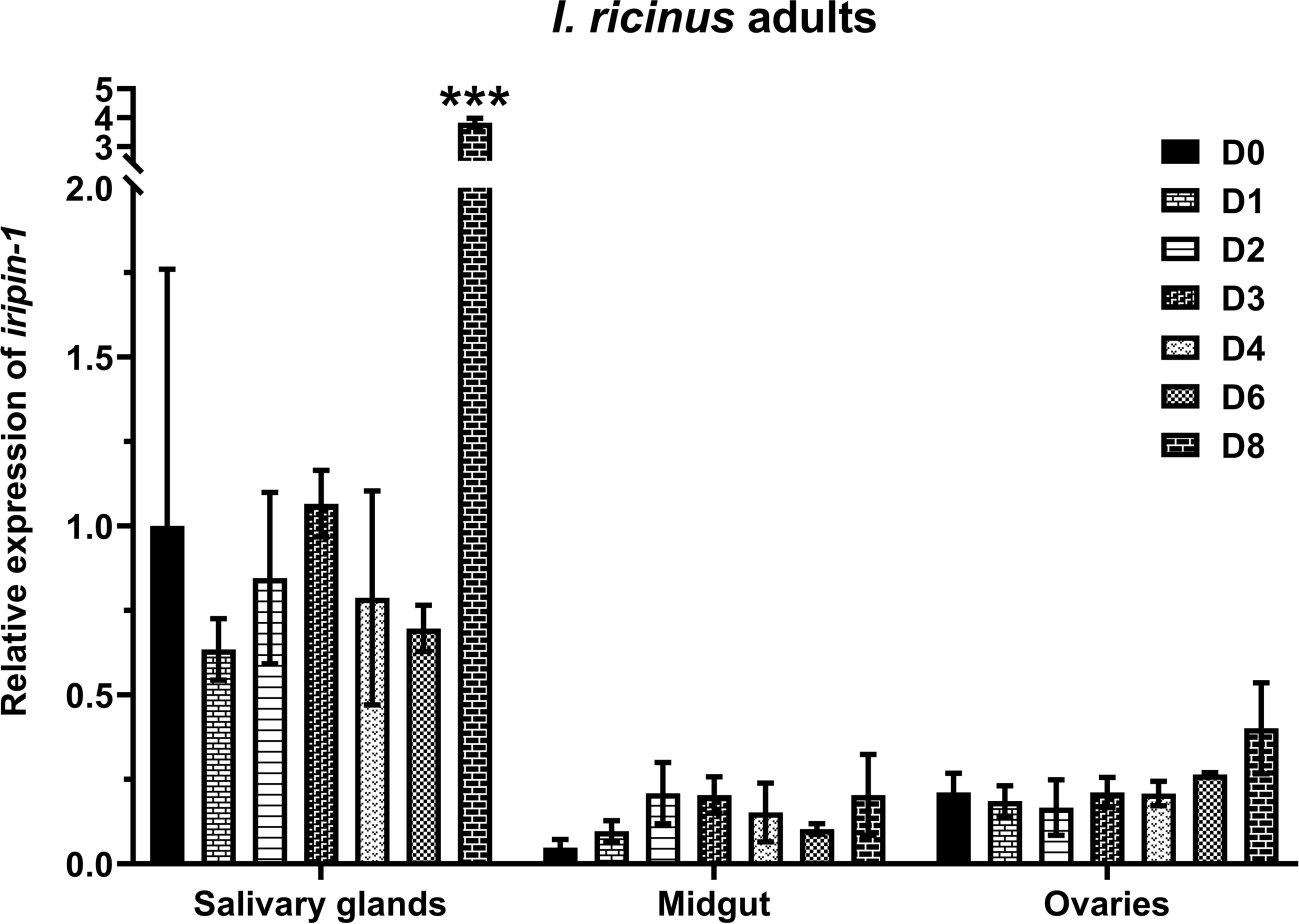
*Iripin-1* is expressed primarily in the salivary glands of adult *I. ricinus* females. Salivary glands, midguts, and ovaries removed from unfed ticks (D0) or from ticks feeding on guinea pigs for 1 (D1), 2 (D2), 3 (D3), 4 (D4), 6 (D6), or 8 (D8) days served as a source of RNA for the analysis of *iripin-1* transcription by RT-qPCR. Relative expression values were calculated using the 2^-ΔΔCt^ (Livak) method (33) and normalized to the reference gene *rps4*. Salivary glands of unfed (D0) ticks were used as a calibrator during calculations, and their expression value was set to 1. Data are presented as mean of three biological replicates ± the standard error of the mean (SEM). The statistically significant increase of *iripin-1* expression in the salivary glands of ticks feeding for 8 days as compared to the salivary glands of unfed ticks is marked with three asterisks (p < 0.001).

In order to see how *iripin-1* transcription changes in adult ticks during blood feeding, we performed RT-qPCR analysis with RNA extracted from salivary glands, midguts, and ovaries of *I. ricinus* females that were either unfed or were feeding on a host for 1, 2, 3, 4, 6, and 8 days. *Iripin-1* mRNA was expressed more abundantly in the salivary glands than in the midguts or ovaries of adult ticks. Significant upregulation of *iripin-1* transcription occurred only in the salivary glands of ticks feeding for 8 days (**Figure 1**). These results suggest that Iripin-1 probably does not play an important role in blood meal digestion or tick reproduction but instead might facilitate blood acquisition by modulating the host defensive response, especially during the late, rapid feeding phase.

### 3.2 Iripin-1 is a potentially secreted serpin with inhibitory function

**Figure 2:**
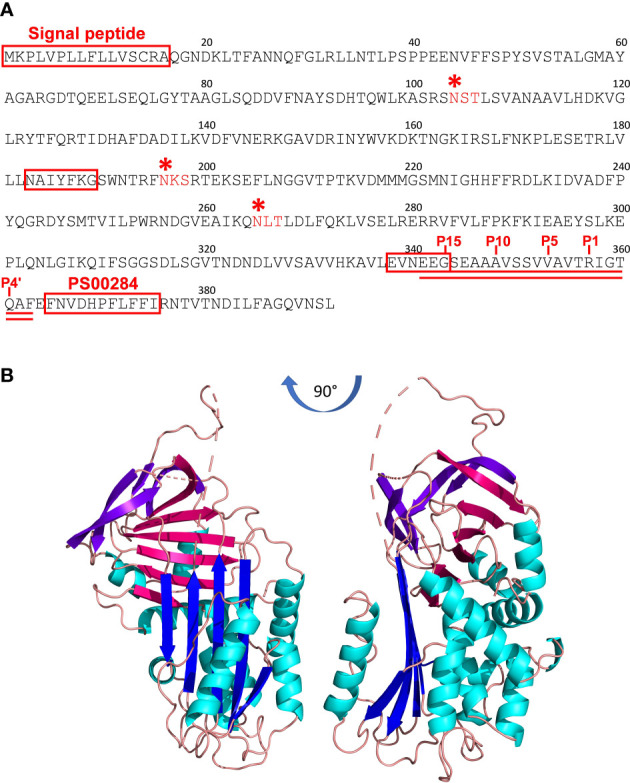
The primary and tertiary structure of Iripin-1. **(A)** The amino acid sequence of Iripin-1. The signal peptide at the N-terminus of the sequence, serpin signature motif PS00284, and serpin consensus amino acid motifs NAIYFKG and EVNEEG are boxed in red. Three predicted N-linked glycosylation sites are marked with red asterisks. The RCL is double underlined. The amino acid residues in the RCL region are numbered based on the standard nomenclature developed by Schechter and Berger (35). According to this nomenclature, the residues on the N-terminal side of the scissile (P1–P1’) bond are not primed and those on the C-terminal side are primed. **(B)** Cartoon representation of the tertiary structure of native Iripin-1. The Iripin-1 structure consists of ten α-helices (cyan) and three β-sheets, of which β-sheet A is blue, β-sheet B is magenta, and β-sheet C is purple. Loops are colored salmon. The loop regions of the Iripin-1 structure with missing amino acid residues because of a poor electron density map are depicted as dashes. The RCL can be seen at the top of the molecule where it acts as a bait for target proteases.

The full-length nucleotide sequence of Iripin-1 (previously published as IRS-1) was obtained as described earlier (32) and was submitted to GenBank under accession number DQ915842.1. This 2,028bp-long sequence encodes a mature protein of 376 amino acids with an approximate molecular mass of 42 kDa and a theoretical isoelectric point of 5.88. A 16-amino acid signal peptide was predicted at the N-terminus of the serpin (**Figure 2A**), suggesting Iripin-1 secretion into tick saliva given its dominant expression in the salivary glands (**Figure 1**). The hinge region of Iripin-1 is formed by glycine at the P15 site, serine at the P14 site, glutamic acid at the P13 position, and amino acid residues with short side chains (alanine and valine) at positions P12–P9 (**Figure 2A**), which corresponds to RCLs of inhibitory serpins (36). Since the P1 site is occupied by the basic amino acid arginine (**Figure 2A**), Iripin-1 might inhibit trypsin and trypsin-like serine proteases rather than chymotrypsin-like or elastase-like serine proteases (37, 38). The server NetNGlyc 1.0 predicted three potential N-linked glycosylation sites (N-X-[S/T]) in the amino acid sequence of Iripin-1 (**Figure 2A**).

### 3.3 Iripin-1 has a typical serpin tertiary structure

The tertiary structure of native Iripin-1 was solved *via* X-ray crystallography at 2.10 Å resolution, and atomic coordinates were deposited in the Protein Data Bank under accession code 7QTZ. The crystal belonged to the monoclinic P12_1_1 space group with unit-cell parameters a=48.82, b=91.01, c=95.84 (Å), α=90.0, β=97.53, γ=90.0 (°), and its asymmetric unit contained two molecules of Iripin-1 (chains A and B) with a calculated solvent content of 50.82% (Matthew’s coefficient) (**Supplementary Table 3**). The final Iripin-1 model contains 359 amino acids in the chain A and 351 amino acids in the chain B. The amino acid regions 180Phe–184Arg in both chains, 324Val–336Ser in the chain A, and 326Glu–346Gln in the chain B could not be modelled in the flexible loop regions of Iripin-1 due to their absence in the electron density map. Iripin-1 adopts a typical serpin fold with an N-terminal helical region and a C-terminal β-sheet domain. Its tertiary structure is composed of ten α-helices and three β-sheets (A, B, C) sequentially arranged in the order α1-β1-α2-α3-α4-α5-β2-α6-β3-α7-β4-β5-β6-β7-β8-β9-α8-α9-β10-β11-α10-β12-β13-β14-β15 (**Figure 2B**). The sheet A consists of five β-strands (β2, β3, β4, β11, β12), sheet B of six β-strands (β1, β7, β8, β9, β14, β15), and sheet C of four β-strands (β5, β6, β10, β13) (**Figure 2B**). Iripin-1 in the crystal adopted a native metastable conformation known as the “stressed” state in which the RCL protrudes from the top of the molecule into the surrounding environment (**Figure 2B**). The analysis of the surface electrostatic potential showed that the Iripin-1 RCL and the adjacent area are slightly positively charged (**Supplementary Figure 2**) which means that Iripin-1 might tend to inhibit proteases with electronegative potential in their active site cleft (8).

### 3.4 Iripin-1 RCL is partially conserved among serpins of hard ticks

**Figure 3:**
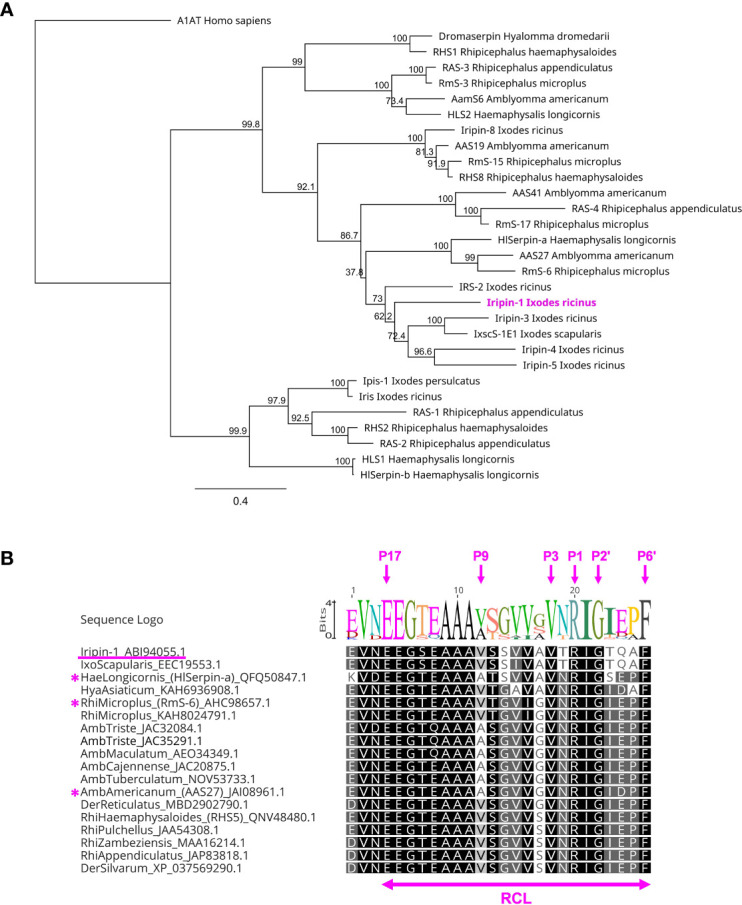
The phylogenetic relationship of Iripin-1 with other tick serpins. **(A)** The phylogenetic tree was built based on the protein sequences of functionally characterized tick serpins using the program PhyML and WAG substitution model. The human serpin α-1-antitrypsin (A1AT) was utilized as an outgroup to root the tree. The length of tree branches represents the number of substitutions per site. The reliability of each clade is expressed by a bootstrap value that was calculated based on 1,000 replicates. **(B)** Alignment of RCL regions of selected tick serpins. The amino acid sequences of serpins with Iripin-1-like RCL were retrieved from GenBank, and their RCLs were aligned using the aligner MUSCLE. The tick species and the GenBank accession number are written next to each sequence. The amino acid residues labeled with magenta arrows are numbered based on the standard nomenclature developed by Schechter and Berger (35). The serpins HlSerpin-a, RmS-6, and AAS27 are marked with a magenta asterisk.

The BLASTP search of the GenBank database of non-redundant protein sequences (nr) identified one Iripin-1 homolog in *Ixodes scapularis* (accession number EEC19553.1) with the amino acid identity of 95.13%. This homologous serpin, however, has not been functionally characterized yet. In the phylogenetic tree, constructed from amino acid sequences of 29 tick serpins with known function, Iripin-1 clustered together with the serpins of ticks *I. scapularis* (IxscS-1E1) and *I. ricinus* (IRS-2, Iripin-3, -4, and -5) (**Figure 3A**). Surprisingly, the comparison of Iripin-1 RCL with the RCLs of other tick serpins indicated that Iripin-1 RCL is partially conserved among the serpins of various ixodid tick species, including the functionally characterized anti-inflammatory serpins HlSerpin-a from *Haemaphysalis longicornis*, RmS-6 from *Rhipicephalus microplus*, and AAS27 from *Amblyomma americanum* (23, 24, 29) (**Figure 3B**). Notably, all RCLs have similar hinge regions (P17–P9) and fully conserved amino acid residues at positions P3 (Val), P1 (Arg), P1’ (Ile), P2’ (Gly), and P6’ (Phe) (**Figure 3B**).

### 3.5 Iripin-1 inhibits trypsin-like serine proteases involved in inflammation

**Figure 4:**
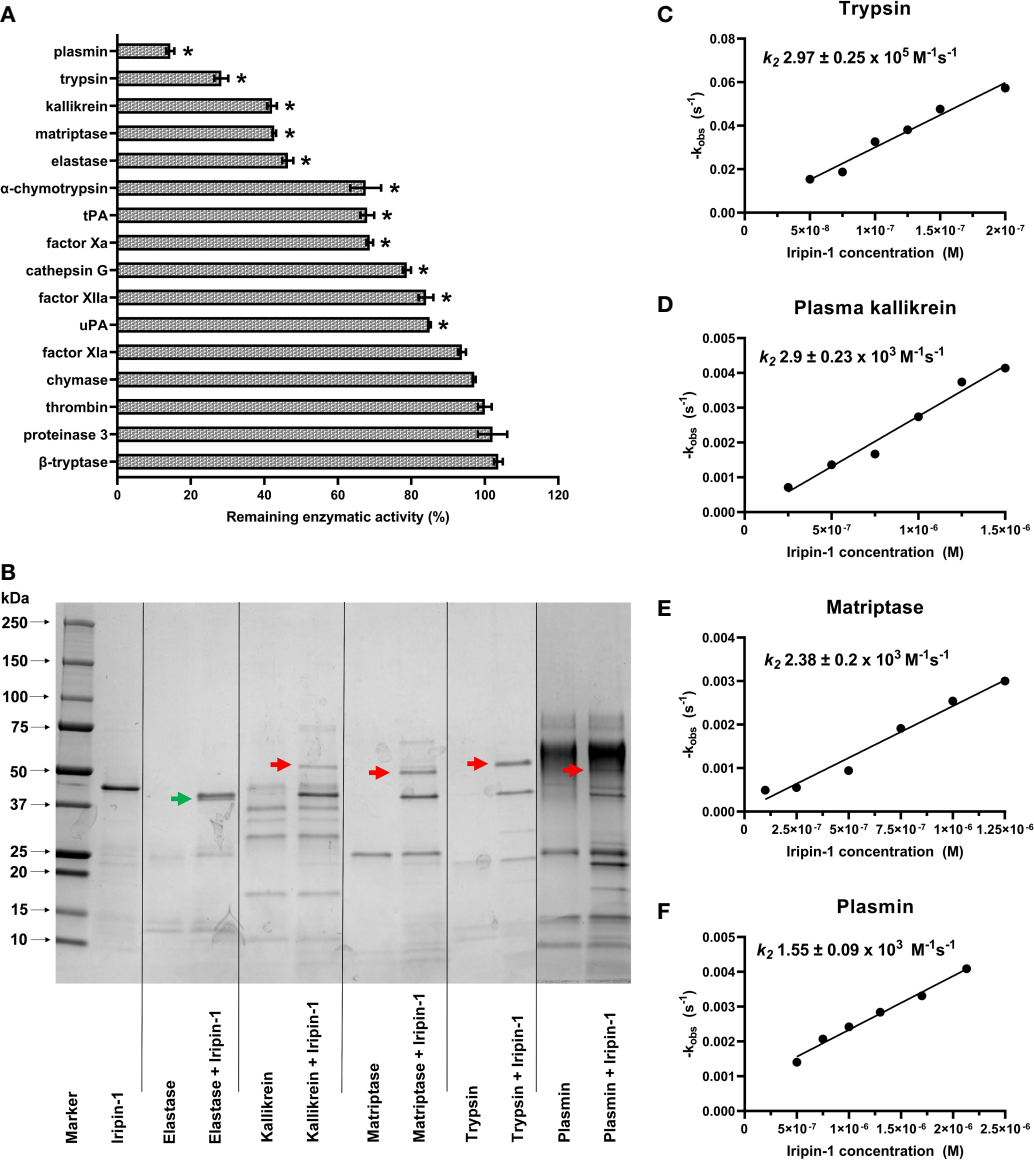
Iripin-1 inhibits trypsin, kallikrein, matriptase, and plasmin *via* a classic serpin inhibitory mechanism. **(A)** The Iripin-1 inhibitory activity against 16 selected serine proteases. The enzymatic activities of individual proteases in the presence of 200 nM Iripin-1 were compared to the substrate hydrolysis rate (set to 100%) in the absence of Iripin-1 and expressed as a percentage. The assay was conducted in triplicate, and data are expressed as mean values ± SEM. Statistically significant reduction (p < 0.05) of proteolytic activity after the incubation of the enzyme with Iripin-1 is marked with an asterisk. tPA, tissue-type plasminogen activator; uPA, urokinase-type plasminogen activator. **(B)** Formation of covalent complexes between Iripin-1 and elastase, kallikrein, matriptase, trypsin, and plasmin. Proteases and Iripin-1 were incubated at equimolar concentrations for 1 h at 37°C, and the presence of complexes was detected by reducing SDS-PAGE. Red arrows point to covalent complexes formed between Iripin-1 and individual proteases, and a green arrow points to Iripin-1 cleaved in its RCL by neutrophil elastase. **(C-F)** Determination of inhibition constants. The apparent first-order rate constant *k_obs_* was plotted against six different Iripin-1 concentrations, and linear regression was performed to obtain the line of best fit. The slope of the line represents the second-order rate constant *k_2_* for the inhibition of trypsin **(C)**, plasma kallikrein **(D)**, matriptase **(E)**, and plasmin **(F)** by Iripin-1. For each determination, the standard error of the slope is given.

To determine the inhibitory specificity of Iripin-1, we firstly tested the capacity of this serpin to reduce the enzymatic activity of 16 selected serine proteases. In this initial screen, Iripin-1 inhibited 11 serine proteases with a statistical significance (**Figure 4A**). However, only the proteolytic activities of plasmin, trypsin, kallikrein, matriptase, and neutrophil elastase were reduced by more than 50% in the presence of Iripin-1 (**Figure 4A**). We proceeded with the evaluation of covalent complex formation between Iripin-1 and the five most strongly inhibited proteases. Iripin-1 formed SDS- and heat-stable complexes with trypsin and trypsin-like serine proteases kallikrein, matriptase, and plasmin (**Figure 4B**). No covalent complex was formed between Iripin-1 and elastase under given experimental conditions (**Figure 4B**), which suggests that the reduced enzymatic activity of elastase observed in the initial screening experiment (**Figure 4A**) is a result of Iripin-1 acting as a substrate for elastase rather than an inhibitor. Finally, we determined the second-order rate constants *k_2_* for Iripin-1 interaction with trypsin, plasma kallikrein, matriptase, and plasmin. Of these four serine proteases, Iripin-1 inhibited most potently trypsin with *k_2_* = 2.97 ± 0.25 × 10^5^ M^-1^ s^-1^ (**Figure 4C**). The inhibition of plasma kallikrein (*k_2_* = 2.9 ± 0.23 × 10^3^ M^-1^ s^-1^, **Figure 4D**) and matriptase (*k_2_* = 2.38 ± 0.2 × 10^3^ M^-1^ s^-1^, **Figure 4E**) was approximately 100 times lower when compared to the inhibition of trypsin. Plasmin was inhibited by Iripin-1 with the lowest potency (*k_2_* = 1.55 ± 0.09 × 10^3^ M^-1^ s^-1^, **Figure 4F**). It is worth noting that the Iripin-1-inhibited proteases, especially plasmin, kallikrein, and trypsin, are involved in the inflammatory response by modulating vasodilation, vascular permeability, immune cell migration, and production of pro-inflammatory cytokines and chemokines (39–42). This fact indicates that Iripin-1 might affect inflammation.

### 3.6 Iripin-1 increases the *in vitro*production of chemokines for neutrophils and monocytes

**Figure 5:**
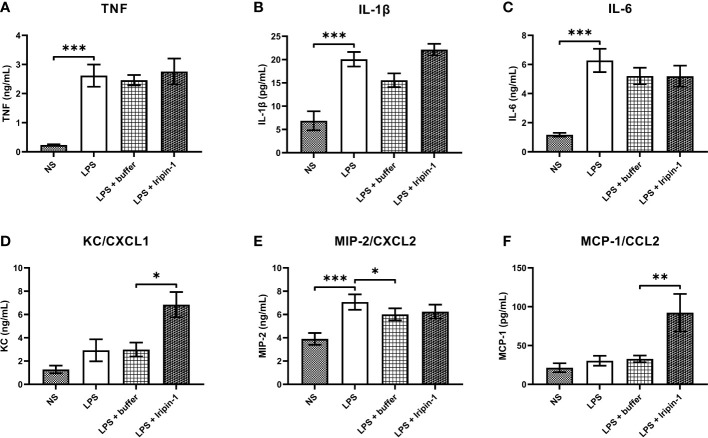
Iripin-1 potentiates the secretion of chemokines for neutrophils (KC) and monocytes (MCP-1). Resident peritoneal cells were treated with 2 μM Iripin-1 or the corresponding amount of control buffer and then were stimulated by LPS (1 μg/mL). Cell-free supernatants, collected 4 h after LPS addition, were utilized for the measurement of concentrations of pro-inflammatory cytokines TNF **(A)**, IL-1β **(B)**, and IL-6 **(C)** and chemokines KC **(D)**, MIP-2 **(E)**, and MCP-1 **(F)**. Data are expressed as mean values ± SEM, and statistically significant differences between the groups NS and LPS, LPS and LPS + buffer, LPS + buffer and LPS + Iripin-1 are marked with asterisks (*p < 0.05, **p < 0.01, ***p < 0.001). The experiment was repeated four times. NS, unstimulated cells.

Since Iripin-1 inhibited serine proteases involved in inflammation, and its RCL is similar to RCLs of tick serpins with known anti-inflammatory activities, we proceeded to test whether Iripin-1 affects the production of pro-inflammatory cytokines and chemokines by resident peritoneal cells (RPCs). Flow cytometric analysis revealed that RPCs, obtained from peritoneal cavities of naïve mice by peritoneal lavage, consisted mainly of B lymphocytes (~ 56%), macrophages (~ 20%), and T lymphocytes (~ 12%) (data not shown). Of these three cell populations, especially resident peritoneal macrophages are known to initiate acute peritoneal inflammation in response to a stimulus by secreting cytokines and chemokines for neutrophils and monocytes (43–45). Indeed, stimulation of RPCs with LPS led to a pronounced increase in the production of pro-inflammatory cytokines TNF, IL-1β, and IL-6 (**Figures 5A–C**) and the neutrophil chemoattractant MIP-2 (**Figure 5E**), but it had only a limited effect on the production of the neutrophil chemokine KC and the monocyte chemotactic factor MCP-1 (**Figures 5D, F**). In comparison with the control (buffer-treated) group, Iripin-1 had no significant effect on the secretion of TNF, IL-1β, IL-6, and MIP-2 (**Figures 5A–C, E**), but it markedly enhanced the production of KC and MCP-1 (**Figures 5D, F**).

### 3.7 Iripin-1 inhibits the recruitment of neutrophils and monocytes to the site of inflammation

**Figure 6:**
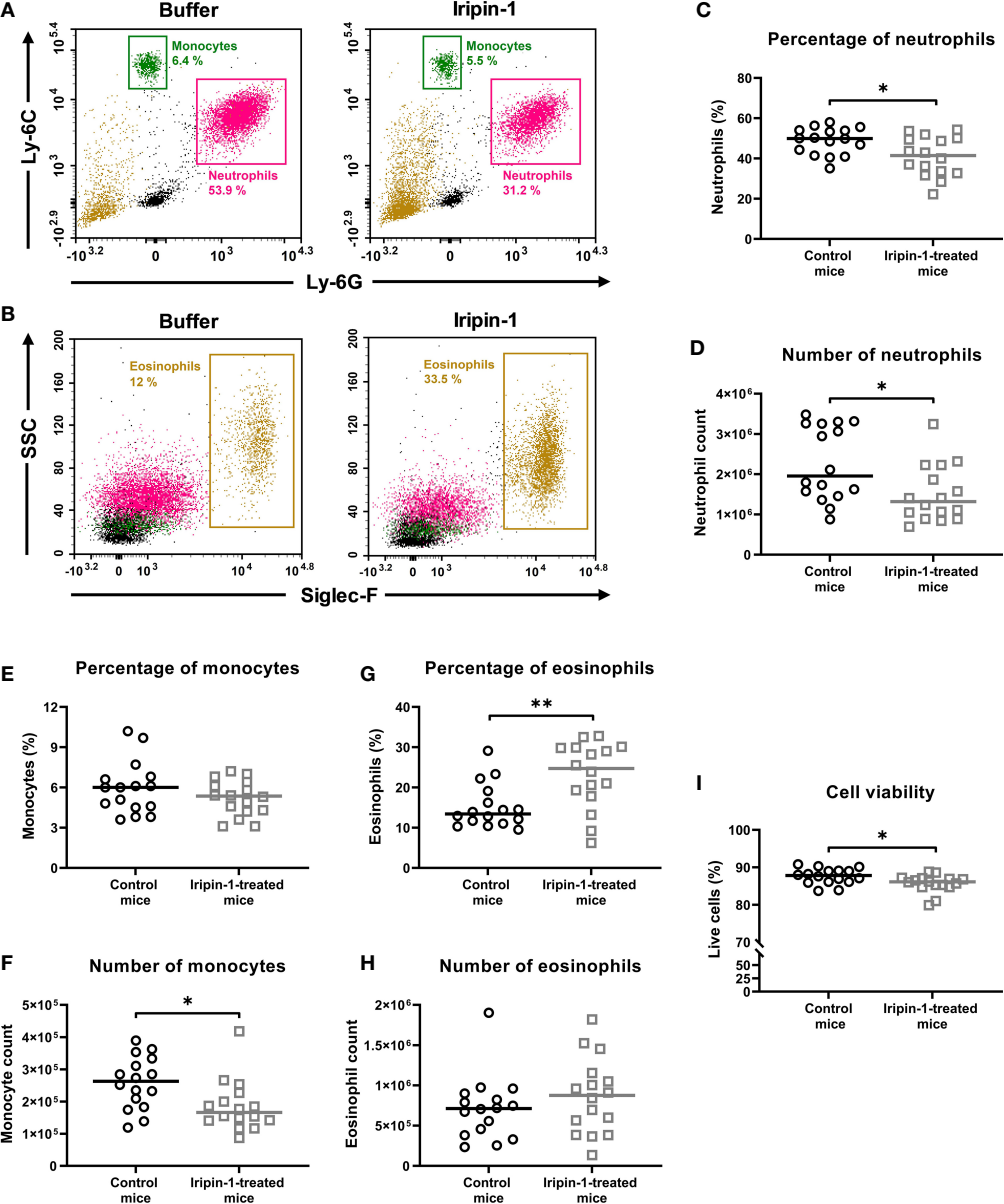
Iripin-1 inhibits the recruitment of neutrophils and monocytes and has no significant effect on the migration of eosinophils in the thioglycolate-induced peritonitis model. **(A, B)** Dot plots showing the percentage of **(A)** neutrophils (Ly-6G^+^ Ly-6C^+^ cells), monocytes (Ly-6C^high^ cells), and **(B)** eosinophils (Siglec-F^+^ cells) in the peritoneal cavities of mice injected with either control buffer and TGM or Iripin-1 and TGM. SSC, side scatter. **(C, E, G, I)** Mice were injected ip with Iripin-1 (2 mg/kg) or the corresponding amount of control buffer, and peritonitis was induced with ip injection of 200 μL 3% TGM. Cells obtained by peritoneal lavage 4 h after TGM administration were analyzed on a flow cytometer to determine the percentages of neutrophils **(C)**, monocytes **(E)**, and eosinophils **(G)** among all live cells, and the percentage of viable cells **(I)** among single cells. **(D, F, H)** Cells harvested by peritoneal lavage were also counted under a light microscope, and the combination of flow cytometry data with known cell numbers provided the information about the numbers of neutrophils **(D)**, monocytes **(F)**, and eosinophils **(H)** that migrated to inflamed peritoneal cavities. In the graphs, each control mouse is represented by one black-lined circle, and each Iripin-1-treated mouse is represented by one grey-lined square. The black and grey horizontal lines mark the median values. Asterisks indicate statistically significant difference between the two groups (*p < 0.05, **p < 0.01).

Using the thioglycolate-induced peritonitis model, we next investigated how Iripin-1 affects the migration of neutrophils, monocytes, and eosinophils *in vivo*. Both the percentage and the number of neutrophils were significantly decreased in Iripin-1-treated mice when compared to control mice (**Figures 6A, C, D**). Monocyte numbers were also reduced in the peritoneal cavities of Iripin-1-injected mice even though the percentages of monocytes did not significantly differ between the two mouse groups (**Figures 6A, E, F**). In contrast to neutrophils and monocytes, the percentage of eosinophils was markedly increased in the peritoneal lavage fluid of Iripin-1-treated mice when compared to control mice, but the eosinophil number was not significantly affected by Iripin-1 administration (**Figures 6B, G, H**). Therefore, the increase in the proportion of eosinophils is a consequence of decreased neutrophil and monocyte numbers in Iripin-1-treated peritoneal cavities rather than enhanced eosinophil migration. Since the neutrophil, monocyte and eosinophil percentage was calculated from live cells only, impaired cell survival might also contribute to the decrease in cell numbers. The viability of cells derived from Iripin-1-treated mice was indeed statistically significantly reduced, yet the difference in the mean percentage of live cells between the control group (87.6%) and the Iripin-1 group (85.7%) is negligible (**Figure 6I**). Thus, the observed significant drop in neutrophil and monocyte numbers is not caused by increased cell death in the presence of Iripin-1. Overall, it can be concluded that Iripin-1 inhibits the migration of neutrophils and monocytes and has no significant effect on the recruitment of eosinophils to the site of inflammation.

### 3.8 Iripin-1 enhances the production of chemokines and anti-inflammatory cytokine IL-10 *in vivo*

**Figure 7:**
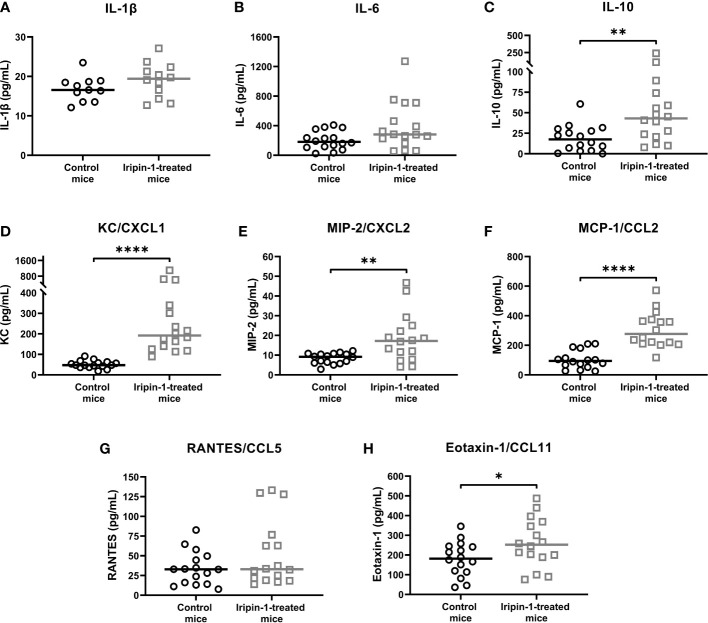
Iripin-1 alters cytokine and chemokine production in the thioglycolate-induced peritonitis model. Mice were injected ip with Iripin-1 (2 mg/kg) or the corresponding amount of control buffer, and peritonitis was induced with ip injection of 200 μL 3% thioglycolate medium (TGM). Peritoneal lavage was performed 4 h after TGM administration, and concentrations of pro-inflammatory cytokines IL-1β **(A)** and IL-6 **(B)**, the anti-inflammatory cytokine IL-10 **(C)**, and chemokines KC **(D)**, MIP-2 **(E)**, MCP-1 **(F)**, RANTES **(G)**, and eotaxin-1 **(H)** in the peritoneal lavage fluid were determined by sandwich ELISA kits. Each control mouse is represented by one black-lined circle, and each Iripin-1-treated mouse is represented by one grey-lined square. The black and grey horizontal lines mark the median values. Asterisks indicate statistically significant differences between the two groups (*p < 0.05, **p < 0.01, ****p < 0.0001).

As Iripin-1 significantly potentiated the secretion of KC and MCP-1 by resident peritoneal cells *in vitro*, we further tested whether this serpin alters cytokine and chemokine production in the thioglycolate-induced peritonitis model. The production of pro-inflammatory cytokines IL-1β and IL-6 in the peritoneal cavities of mice injected with thioglycolate medium was not significantly affected by Iripin-1 administration (**Figures 7A, B**), and the level of TNF was below the detection limit of the ELISA kit used. In contrast to the pro-inflammatory cytokines, the concentration of the anti-inflammatory cytokine IL-10 was significantly increased in Iripin-1-treated mice when compared to control mice (**Figure 7C**). Of the chemokines detected in the peritoneal lavage fluid, Iripin-1 exhibited the strongest enhancing effect on the production of the neutrophil chemotactic factor KC and the monocyte chemoattractant MCP-1 (**Figures 7D, F**), which is consistent with the results of the *in vitro* experiment with resident peritoneal cells (**Figures 5D, F**). The secretion of the neutrophil chemokine MIP-2 and the eosinophil chemoattractant eotaxin-1 was also slightly enhanced in response to Iripin-1 injection (**Figures 7E, H**). The only chemokine with unaltered production in the presence of Iripin-1 was RANTES (**Figure 7G**).

### 3.9 Iripin-1 modifies the expression of Mac-1 and CXCR2 on the surface of neutrophils

**Figure 8:**
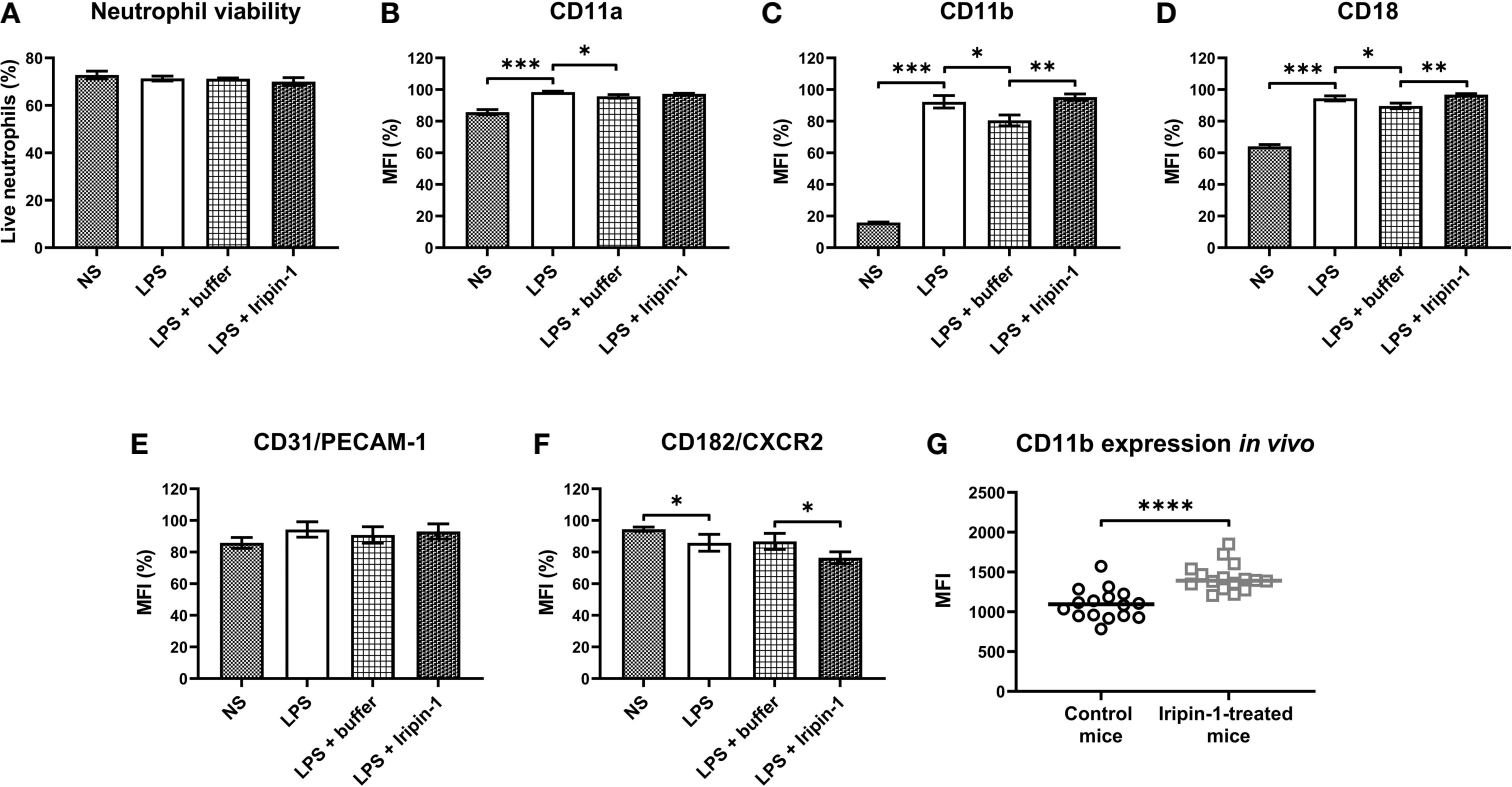
Iripin-1 potentiates the expression of Mac-1 (CD11b/CD18) and reduces the expression of CXCR2 on the neutrophil surface. **(A-F)** Neutrophils obtained by magnetic-activated cell sorting of bone marrow cells were cultured in the presence of LPS (1 μg/mL), control buffer, and Iripin-1 (2 μM) for 3 h and subsequently the cell viability **(A)** and expression levels of CD11a **(B)**, CD11b **(C)**, CD18 **(D)**, PECAM-1 **(E)**, and CXCR2 **(F)** were determined using multi-color flow cytometry. Data in the graphs are expressed as mean values of three repetitions of the experiment ± SEM. The graph in **(A)** shows the percentage of viable neutrophils among single cells. The median fluorescence intensity (MFI) values in **(B–F)** are expressed as a percentage of the highest MFI value (which was set to 100%) measured in each repetition of the experiment. Statistically significant differences between the groups NS and LPS, LPS and LPS + buffer, LPS + buffer and LPS + Iripin-1 are marked with asterisks (*p < 0.05, **p < 0.01, ***p < 0.001). NS, unstimulated cells. **(G)** Mice were injected ip with Iripin-1 (2 mg/kg) or the corresponding amount of control buffer, and peritonitis was induced with ip injection of 200 μL 3% TGM. Cells obtained by peritoneal lavage 4 h after TGM administration were analyzed on a flow cytometer to determine the expression level of CD11b on the neutrophil surface. Each control mouse is represented by one black-lined circle, and each Iripin-1-treated mouse is represented by one grey-lined square. The black and grey horizontal lines mark the median values. Asterisks indicate statistically significant difference between the two groups (****p < 0.0001). MFI, median fluorescence intensity.

Previous experiments showed that Iripin-1 inhibits the recruitment of neutrophils and monocytes to the site of inflammation despite enhanced chemokine secretion. One way that Iripin-1 might mediate the inhibition of cell migration in the presence of increased chemokine concentrations would be the modification of the expression of chemokine receptors, e.g., CXC chemokine receptor-2 (CXCR2). Moreover, Iripin-1 might alter the surface expression of molecules involved in the firm adhesion of leukocytes to endothelial cells and subsequent transendothelial migration, such as LFA-1, Mac-1, and PECAM-1. Therefore, the aim of the next experiment was to assess whether Iripin-1 modulates the expression of the molecules mentioned above on the surface of LPS-stimulated bone marrow-derived neutrophils. Incubation in the presence of LPS, control buffer, and Iripin-1 had no effect on neutrophil viability (**Figure 8A**). In comparison with buffer-treated cells, Iripin-1 did not significantly alter the expression of molecules LFA-1 (CD11a/CD18) and PECAM-1 (**Figures 8B, E**), but it slightly reduced the expression of CXCR2, a receptor for chemokines KC and MIP-2 (**Figure 8F**). Besides decreasing the CXCR2 level, Iripin-1 also significantly increased the expression of the integrin Mac-1 (CD11b/CD18) (**Figures 8C, D**). Iripin-1-mediated enhancement in the number of CD11b molecules on the neutrophil surface was also observed in the thioglycolate-induced peritonitis model (**Figure 8G**), which suggests that the *in vitro* results correspond to what is happening *in vivo*. Based on the outcomes of this *in vitro* experiment, we conclude that the recruitment of neutrophils to the site of inflammation might be inhibited due to the Iripin-1-mediated decrease in the expression of CXCR2 on neutrophil surfaces.

### 3.10 Iripin-1 decreases the expression of certain cell adhesion molecules on the surface of HUVECs

**Figure 9:**
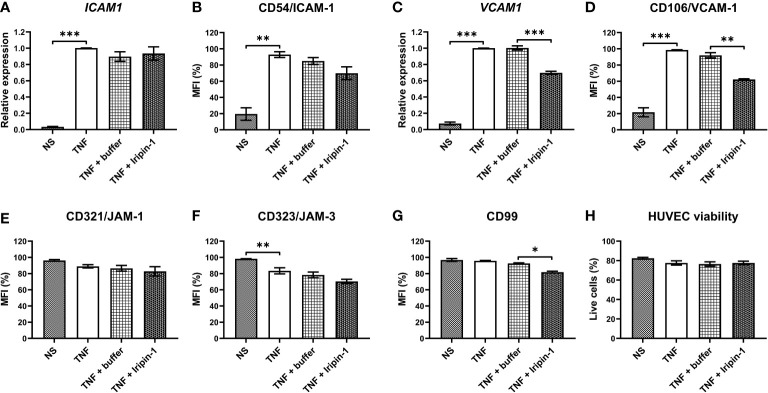
Iripin-1 modifies the expression of cell adhesion molecules in TNF-stimulated HUVECs. **(A, C)** HUVEC incubation in the absence of TNF (NS, unstimulated cells) or in the presence of TNF (10 ng/mL), Iripin-1 (2 μM), and control buffer for 4 h was followed by the isolation of total RNA and RT-qPCR analysis to determine the expression level of *ICAM1* **(A)** and *VCAM1* **(C)**. Relative expression values were calculated using the Pfaffl method (46) and a combination of two reference genes *GAPDH* and *HPRT1*. Cells treated with TNF only were used as a calibrator during calculations. **(B, D-H)** HUVECs were cultured in the absence of TNF (NS, unstimulated cells) or in the presence of TNF (10 ng/mL), Iripin-1 (2 μM), and control buffer for 6 h and subsequently the cell surface expression of ICAM-1 **(B)**, VCAM-1 **(D)**, JAM-1 **(E)**, JAM-3 **(F)**, and CD99 **(G)** together with HUVEC viability **(H)** were assessed by multicolor flow cytometry. The graphs in **(B)** and **(D-G)** show the median fluorescence intensity (MFI) values expressed as a percentage of the highest MFI value (which was set to 100%) measured in each repetition of the experiment, and the graph in **(H)** shows the proportion of viable HUVECs among single cells. Data in all graphs **(A-H)** are depicted as mean values of three repetitions of the experiment ± SEM. The statistically significant differences between the groups NS and TNF, TNF and TNF + buffer, TNF + buffer and TNF + Iripin-1 are marked with asterisks (*p < 0.05, **p < 0.01, ***p < 0.001).

Besides investigating the effect of Iripin-1 on the expression of molecules on the surface of neutrophils, we likewise tested whether this tick serpin modulates the expression of cell adhesion molecules in TNF-stimulated HUVECs by employing flow cytometry and RT-qPCR. As with neutrophils, we mainly focused on the proteins involved in the firm adhesion of leukocytes to endothelial cells and extravasation across the blood vessel wall, namely ICAM-1, VCAM-1, CD99, JAM-1, and JAM-3. The *ICAM1* mRNA level was not affected by Iripin-1 treatment, but ICAM-1 protein expression on the surface of Iripin-1-treated HUVECs was reduced when compared to buffer-treated cells (**Figures 9A, B**). Unlike ICAM-1, the expression of VCAM-1 was markedly attenuated in the presence of Iripin-1 at both mRNA and protein levels (**Figures 9C, D**). The protein level of JAM-1 and JAM-3 on the surface of HUVECs was not significantly altered by Iripin-1 (**Figures 9E, F**), but the serpin decreased the expression of the transmembrane protein CD99 (**Figure 9G**). The viability of HUVECs was not impaired in response to Iripin-1 treatment (**Figure 9H**). Overall, Iripin-1-mediated reduction in the expression of cell adhesion molecules on the surface of endothelial cells (especially VCAM-1 and CD99) might contribute to the inhibition of neutrophil and monocyte recruitment to the site of inflammation.

## 4 Discussion

Tick saliva contains hundreds to thousands of non-proteinous molecules and proteins belonging to diverse protein families that allow ticks to feed on a host for several days due to the maintenance of blood fluidity and suppression of host’s immune and wound healing responses (20, 47). Since tick salivary components have been shown to possess anticoagulant, anti-inflammatory, anti-complement, and immunomodulatory activities, it is not surprising that tick saliva is considered to be a rich source of bioactive molecules with therapeutic potential in the treatment of hypercoagulable states, inflammatory diseases, complement-related disorders, and cancer (21, 48, 49). Besides the development of pharmaceuticals, tick salivary proteins might be utilized as components of anti-tick vaccines that would disrupt tick feeding and limit or even block the transmission of multiple tick-borne pathogens (50, 51). For the efficient selection of suitable candidates for drug or anti-tick vaccine development, it is necessary to know the structure and function of as many tick proteins as possible. In this study, we report on the structural and functional characterization of *I. ricinus* serpin Iripin-1.

The tertiary structure of Iripin-1 in its native metastable state resembles 3D structures of other native serpins, including *I. ricinus* serpins Iripin-4 and Iripin-8 (52). The most striking difference among the serpins is the length of RCL. The RCL of Iripin-1 has a similar length as Iripin-4 RCL and is 5 amino acid residues shorter than Iripin-8 RCL (**Supplementary Figure 2**). Despite similar tertiary structures, the three serpins differ in the distribution of electrostatic potential on their surfaces, including RCLs (**Supplementary Figure 2**). While Iripin-1 and Iripin-8 have a positively charged RCL, Iripin-4 RCL is slightly electronegative. Distinctions in surface electrostatic potential might contribute to the differential ability of serpins to inhibit various proteases as well as bind co-factors and/or other ligands (8, 53).

The amino acid composition of the hinge region indicated that Iripin-1 is an inhibitory serpin, and the presence of arginine at the P1 site of the RCL further implied that this serpin could primarily target trypsin-like serine proteases. This was confirmed experimentally when Iripin-1 formed covalent complexes with trypsin and trypsin-like serine proteases kallikrein, matriptase, and plasmin. On the contrary, no covalent complex was formed between Iripin-1 and elastase under given experimental conditions. The absence of the complex might be caused by the fact that elastase prefers aliphatic amino acids valine, alanine, or isoleucine at the P1 position of its substrates (54). Incompatibility in local surface electrostatic potential might also contribute to Iripin-1 acting as a substrate for elastase rather than an inhibitor. The active site of elastase is less negatively charged than the active site clefts of kallikrein, matriptase, plasmin, and trypsin (**Supplementary Figure 3**). Therefore, Iripin-1, possessing electropositive RCL and adjacent areas, might react less efficiently with elastase than with the other proteases (8).

RT-qPCR analysis showed that Iripin-1 is mainly expressed in the salivary glands of *I. ricinus* adults. Since the signal peptide was predicted at the N-terminus, Iripin-1 is most likely secreted *via* saliva into the feeding cavity where it contributes to the modulation of the host’s defensive response. In the protease inhibition assay, Iripin-1 had no effect on the proteolytic activity of thrombin and blood clotting factor XIa and only slightly decreased the activity of factors Xa and XIIa. In accordance with these results, Iripin-1 did not significantly prolong plasma clotting time in prothrombin time (PT), activated partial thromboplastin time (aPTT), and thrombin time (TT) coagulation assays (data not shown). These findings suggest that Iripin-1 does not target the host’s coagulation cascade. In fact, the Iripin-1’s ability to inhibit trypsin, kallikrein, and plasmin indicates that this serpin suppresses the inflammatory response rather than blood coagulation. The amino acid composition of the RCL also supports the anti-inflammatory role of Iripin-1. Three functionally characterized tick serpins, AAS27, HlSerpin-a, and RmS-6, have a similar arrangement of amino acids in their RCLs as Iripin-1, with the basic amino acid residue arginine at the P1 site. Similarly to Iripin-1, both AAS27 and RmS-6 inhibited trypsin and plasmin, while the inhibitory activity of HlSerpin-a against these two serine proteases has not been tested yet (23, 29, 55). Interestingly, none of these three serpins has been shown to suppress blood coagulation, but all of them exerted anti-inflammatory activities either *in vitro* or *in vivo*. AAS27 attenuated formalin- and compound 48/80-induced acute inflammation in rats (23). HlSerpin-a suppressed the production of pro-inflammatory cytokines TNF and IL-6 by LPS-stimulated macrophages and dendritic cells and relieved inflammation in a murine arthritis model (29). Finally, RmS-6 reduced vascular permeability in rat skin induced by formalin injection (24).

Since Iripin-1 primarily inhibits proteases involved in inflammation (39, 41, 42), and its RCL resembles RCLs of tick serpins with proved anti-inflammatory activities, we further tested the effect of Iripin-1 on cytokine and chemokine production and immune cell migration. Iripin-1 did not significantly alter the production of pro-inflammatory cytokines TNF, IL-1β, and IL-6, but increased IL-10 secretion *in vivo*. Elevated concentration of IL-10 at the feeding site should be beneficial for ticks as this cytokine suppresses both innate and adaptive immune responses (56). In contrast to no significant effect on the production of pro-inflammatory cytokines, Iripin-1 stimulated the secretion of several chemokines for neutrophils, monocytes, and eosinophils, namely KC/CXCL1, MIP-2/CXCL2, MCP-1/CCL2, and eotaxin-1/CCL11. Increase in the production of chemokines does not seem to be in the ticks’ best interest as elevated chemokine levels would attract immune cells to the site of tick attachment. However, *I. ricinus* saliva is a complex mixture of many proteins, some of which have been shown to inhibit chemokine production (26, 27, 57). Moreover, *I. ricinus* produces evasins, which are glycoproteins that bind and neutralize host’s chemokines, thus inhibiting immune cell recruitment (58, 59). To date, *I. ricinus* evasins have been shown to bind multiple Glu-Leu-Arg (ELR)^+^ and ELR^-^ CXC chemokines, including CXCL1, CXCL2, and CXCL8 (59, 60). Therefore, Iripin-1-stimulated production of “undesirable” chemokines can be inhibited by other proteins in tick saliva, or the cell-attracting activity of these chemokines can be blocked by evasin binding.

Nevertheless, increased secretion of certain chemokines could be beneficial for ticks, especially during the late, rapid feeding phase. Saliva obtained from *I. ricinus* adults that fed on a host for 6-7 days strongly enhanced the production of three chemokines, including MCP-1 (61). Since *iripin-1* expression in the salivary glands of *I. ricinus* ticks was markedly upregulated around the 8^th^ day of blood feeding, this serpin might be responsible for the saliva-mediated increase in MCP-1 secretion. MCP-1 potently triggers degranulation of mast cells and basophils, leading to histamine release (62–64). Since histamine induces vasodilation and enhances vascular permeability (65), its presence at the tick feeding site could increase blood flow, thus facilitating tick engorgement. Indeed, immunization of mice with *I*. *scapularis* histamine release factor (tHRF) impaired tick engorgement in the rapid feeding phase, as evidenced by decreased weight of ticks. Moreover, the injection of histamine into the skin at the tick bite site led to a significant increase in *I. scapularis* weights (66). Therefore, Iripin-1-mediated enhancement of MCP-1 production and subsequent MCP-1-induced histamine release from granules of mast cells and basophils might facilitate *I. ricinus* engorgement in the late, rapid feeding phase.

Iripin-1 administration impaired neutrophil and monocyte recruitment and had no significant effect on eosinophil migration in the thioglycolate-induced peritonitis model. The inhibition of neutrophil migration was likely not caused by Iripin-1-mediated reduction in the expression of cell adhesion molecules on neutrophil surfaces because Iripin-1 did not attenuate the *in vitro* expression of LFA-1, Mac-1, and PECAM-1 by LPS-stimulated neutrophils. On the other hand, the *in vitro* neutrophil assay also showed that Iripin-1 slightly decreases the surface expression of CXCR2, which is a receptor for chemokines KC and MIP-2 (67). If the reduction in CXCR2 expression also occurs *in vivo*, it might result in the impaired migration of neutrophils to the site of inflammation (68). It is intriguing that Iripin-1 inhibited neutrophil and monocyte extravasation and simultaneously substantially increased the production of chemokines for these two cell populations. Enhanced concentrations of circulating chemokines can downregulate the expression of chemokine receptors on immune cells, thus impeding the migration of the cells to the inflammatory site (69). The effect of Iripin-1 on chemokine levels in mouse blood and the expression of chemokine receptors on circulating immune cells during thioglycolate-induced peritonitis will be the subject of future investigation.

Lastly, we tested the effect of Iripin-1 on the expression of cell adhesion molecules on the surface of HUVECs. Unlike ICAM-1 expression, which was only slightly and insignificantly decreased in the presence of the serpin, VCAM-1 expression was markedly reduced at both mRNA and protein levels. ICAM-1 facilitates the firm adhesion of neutrophils, monocytes, and eosinophils to the endothelium, whereas VCAM-1 was claimed to mediate the adhesion of monocytes and eosinophils but not neutrophils (70, 71). However, there is accumulating evidence suggesting that VCAM-1 is involved in neutrophil extravasation as well (72–74). Thus, Iripin-1-mediated decrease in the surface expression of ICAM-1 and especially VCAM-1 might contribute to the impaired recruitment of neutrophils and monocytes to the site of inflammation. Interestingly, eosinophil migration was not inhibited in Iripin-1-treated mice even though these cells are reported to utilize VCAM-1 to adhere to endothelial cells (70, 75). However, it cannot be excluded that eosinophils rely on other molecules besides VCAM-1, such as MAdCAM-1, to adhere to endothelium in the thioglycolate-induced peritonitis model (76). In addition to VCAM-1, Iripin-1 slightly attenuated the surface expression of CD99, which is a heavily glycosylated transmembrane protein that facilitates neutrophil and monocyte transmigration across the endothelial monolayer (77, 78). Since anti-CD99 antibody substantially inhibited the migration of both neutrophils and monocytes in thioglycolate-induced peritonitis (79, 80), decrease in CD99 expression might be another factor responsible for impaired neutrophil and monocyte recruitment in Iripin-1-treated mice.

The *in vitro* enzymatic assays and SDS-PAGE showed that Iripin-1 is an inhibitor of trypsin, kallikrein, matriptase, and plasmin. Trypsin is present not only in the digestive tract but also in skin epithelial cells, splenic monocytes, macrophages, and lymphocytes (81). Plasma kallikrein and plasmin are predominantly synthesized in the liver as inactive precursors (prekallikrein and plasminogen) and subsequently are secreted into circulation. In blood vessels, plasmin(ogen) binds to neutrophils, monocytes, and other cells *via* plasmin(ogen) receptors, whereas prekallikrein forms a complex with high-molecular-weight kininogen (41, 42, 82). Finally, matriptase is a type II transmembrane serine protease that is expressed mainly by epithelial cells but can also be found on the surface of some leukocytes, including monocytes (83–85). Trypsin, plasmin, and matriptase could facilitate immune cell migration in the extracellular matrix (ECM) through direct degradation of ECM proteins as well as through activation of ECM-degrading matrix metalloproteinases (86–90). Furthermore, plasmin can support cell migration due to its fibrinolytic activity as fibrin(ogen) deposition was noted at the inflammatory site (91). Kallikrein and matriptase can further stimulate fibrinolysis by converting plasminogen to plasmin (92) and by activating urokinase-type plasminogen activator (93, 94). Besides its role in fibrinolysis, kallikrein is responsible for the release of the potent inflammatory mediator bradykinin which triggers vasodilation and increases vascular permeability, thus facilitating immune cell recruitment (95). Kallikrein together with trypsin can also upregulate the expression of ICAM-1 and VCAM-1 on endothelial cells (96, 97). Finally, plasmin-mediated cleavage of MCP-1/CCL2 increases chemotactic potency of this chemokine (98). Since trypsin, kallikrein, matriptase, and plasmin clearly affect various aspects of the cell recruitment process, Iripin-1 might impair immune cell migration to the site of inflammation not only by the modulation of the expression of cell adhesion molecules on endothelium but also by the reduction of the enzymatic activity of serine proteases.

## 5 Conclusion

The structure and function of *I. ricinus* salivary serpin Iripin-1 were determined in this study. The serpin inhibited neutrophil and monocyte migration but had no significant effect on the recruitment of eosinophils in the mouse model of acute peritonitis. The migration of neutrophils and monocytes was not impaired because of reduced chemokine secretion. Based on the results of *in vitro* experiments, we speculate that the recruitment of inflammatory cells might be attenuated *via* Iripin-1-mediated decrease in the expression of cell adhesion molecules on the surface of endothelial cells, modulation of surface expression of chemokine receptors, and/or inhibition of the enzymatic activity of trypsin, kallikrein, matriptase, and plasmin. It is also possible that some additional factors could impede cell migration. Thus, more research is needed to validate the results of *in vitro* experiments in *in vivo* models of inflammation and to identify the exact cause(s) of impaired neutrophil and monocyte recruitment. Moreover, Iripin-1 could be tested in RNA interference and/or vaccination experiments to assess its potential for anti-tick vaccine development.

## Data availability statement

The original contributions presented in the study are included in the article/**Supplementary Material**. Further inquiries can be directed to the corresponding author.

## Ethics statement

The animal study was reviewed and approved by Czech Academy of Sciences (experimental project No. 098/2020) and Ministry of Education, Youth and Sports of the Czech Republic (experimental project No. 19085/2015-3).

## Author contributions

AC designed and performed experiments, analyzed data, and wrote the manuscript. BK, JK, HL, and MK designed and performed experiments and analyzed data. IKS and LT supervised the study. JC supervised the study, designed experiments, and revised the manuscript. All authors contributed to the article and approved the submitted version.
