# Supplementary material for: Iripin-1, a new anti-inflammatory tick serpin, inhibits leukocyte recruitment in vivo while altering the levels of chemokines and adhesion molecules
Source: Front Immunol. 2023 Jan 23;14:1116324. doi: 10.3389/fimmu.2023.1116324 (PMC9901544; doi:10.3389/fimmu.2023.1116324)
Supplement: Supplementary file 1 [file DataSheet_1.pdf]

Supplementary Material

Materials and Methods

Bioinformatics analyses

The molecular weight and isoelectric point (pI) of Iripin-1 were estimated using the program ProtParam (1). The SignalP 4.1 server was utilized for the detection of the signal peptide (2). The serpin signature motif PS00284 and serpin consensus amino acid motifs N-[AT]-[VIM]-[YLH]-F-[KRT]-[GS] and [DERQ]-[VL]-[NDS]-E-[EVDKQ]-G (3) were localized in the protein sequence with the help of the ScanProsite tool (4). Potential N-linked glycosylation sites were predicted using the server NetNGlyc 1.0 (5). The amino acid sequences of Iripin-1's homologs and serpins with RCL regions similar to the Iripin-1 RCL were retrieved from GenBank's Non-redundant protein sequences (nr) and Transcriptome Shotgun Assembly proteins (tsa_nr) with the help of BLASTP (6). The hinge regions and RCLs of retrieved serpins were aligned in the program Geneious Prime 2022.0.2 (Biomatters, Auckland, New Zealand) using MUSCLE 3.8.425 (7).

Production of recombinant Iripin-1

The *iripin-1* sequence without a signal peptide and with an ATG codon inserted into its 5'-terminus was cloned into the pET-17b vector (Novagen, MilliporeSigma, Burlington, MA, USA), and the resulting plasmid was transformed into BL21(DE3)pLysS chemically competent *Escherichia coli* cells (Invitrogen, Waltham, MA, USA). Bacterial cells were grown in LB medium containing ampicillin (50 μg/mL) at 37 °C, and when the OD600 of the culture reached 0.8, 1 mM isopropyl β-D-1-thiogalactopyranoside (IPTG) was added to induce Iripin-1 expression. Purification of the recombinant protein and endotoxin removal were done by the company ARVYS Proteins (Trumbull, CT, USA). Cells harvested after 5 h growth in the presence of IPTG were sonicated 4 × 30 sec in the lysis buffer (50 mM sodium phosphate, pH 7.5 and 500 mM NaCl, supplemented with Benzonase nuclease, lysozyme, and cOmplete protease inhibitor cocktail from Roche), and the lysate was clarified by centrifugation at 40,000 g for 30 min. Washed inclusion bodies containing Iripin-1 were dissolved in 20 mM HEPES, pH 8, 8 M guanidine-HCl, 5 mM dithiothreitol, and 1 mM EDTA, and the inclusion bodies extract diluted to 6 M guanidine-HCl was purified by size exclusion chromatography using a HiPrep 26/60 Sephacryl S-300 HR gel filtration column (Cytiva, Marlborough, MA, USA). Fractions from gel filtration in 20 mM HEPES, pH 8, 4 M guanidine-HCl, 5 mM dithiothreitol, and 1 mM EDTA were pooled and diluted 8 times in refolding buffer (PBS, pH 7.4, 100 mM betaine, 1 mM EDTA, and 12% glycerol). Following incubation for 2 days at 4 °C, the refolded Iripin-1 was concentrated and dialyzed into PBS, pH 7.4, 100 mM betaine, 1 mM EDTA, and 10% glycerol. Endotoxin was removed by a detergent-based method.

Crystallization

An appropriate Iripin-1 concentration for crystallization screening (0.93 mg/mL) was determined by the PCT Pre-Crystallization Test (Hampton Research, Aliso Viejo, CA, USA). Crystallization

experiments were performed using the sitting-drop vapor diffusion technique in Swissci 96-well 2-drop MRC crystallization plates (Molecular Dimensions, Newmarket, UK) with the help of the OryxNano protein crystallization robot (Douglas Instruments, Hungerford, UK). Screening for suitable crystallization conditions was conducted using commercially available crystallization kits. Two protein-to-well solution ratios 1:0.5 and 1:1 (1 µl : 0.5 µl and 1 µl : 1 µl) were applied, and drop solution was equilibrated against 50 µl reservoir solution at 4 °C. The best crystallization condition (No. 1-35, 0.02 M Nickel(II) sulfate hexahydrate pH 7.0, 0.01 M HEPES, 33% v/v Jeffamine M-600) was identified in the MemGold HT-96 screen (Molecular Dimensions).

X-ray data collection, crystal structure determination, and refinement

Before measurements, obtained crystals (**Supplementary Figure 1**) were flash-frozen in liquid nitrogen without any additional cryoprotection. Collection of X-ray diffraction data was performed at the BESSY II electron storage ring on the beamline BL14.2 operated by the Helmholtz-Zentrum Berlin (8). Acquired data were processed using the XDS program package (9) with the XDSAPP graphical user interface (10). Programs that are part of the CCP4 suite (11) were utilized for crystal structure determination and refinement. The scaling was performed in the programs POINTLESS and SCALA (12). The structure of Iripin-1 was solved by the molecular replacement method using MOLREP (13) and the automated molecular replacement pipeline BALBES (14). The crystal structure of human squamous cell carcinoma antigen 1 (Protein Data Bank code 2ZV6) (15) with 35.54% sequence identity and 46.15% similarity to Iripin-1 was utilized as a starting model. The Iripin-1 structure was subsequently refined with the program REFMAC5 (16) and manually rebuilt in Coot (17). The MolProbity server (18) and OneDep system (19) were used for the qualitative validation of the final model. The figures of the Iripin-1 structure were prepared using the molecular visualization system PyMOL (Schrödinger, New York, NY, USA). Data collection and refinement statistics are summarized in **Supplementary Table 3**.

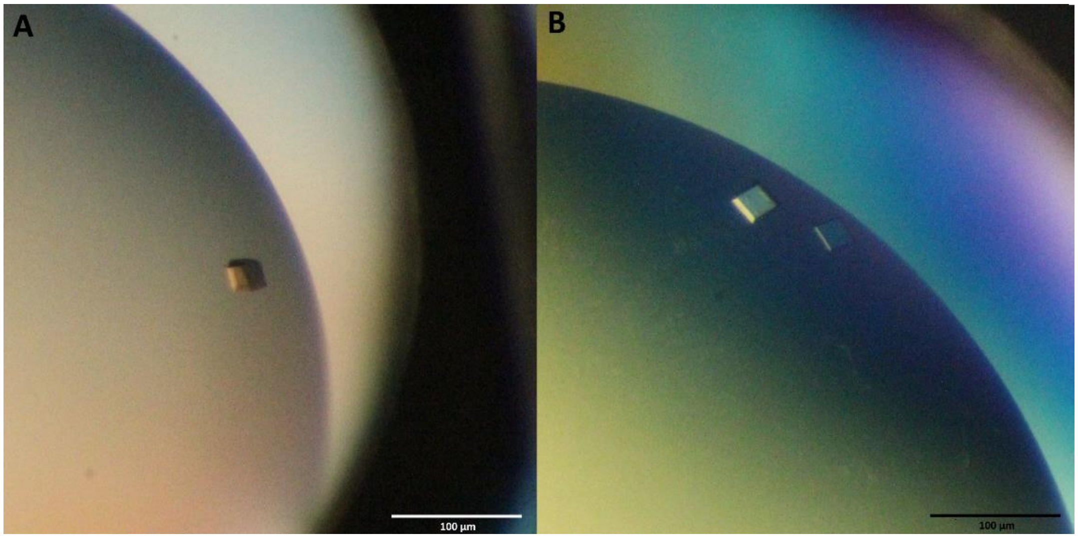

Supplementary Figure 1. The crystals of Iripin-1. (A) The crystal grown in 0.02 M Nickel(II) sulfate hexahydrate pH 7.0, 0.01 M HEPES, 33% v/v Jeffamine M-600, using the protein-to-well solution ratio 1:1 (1 µl : 1 µl). **(B)** The crystals grown under the same conditions as the crystal in **(A)**, using the protein-to-well solution ratio 1:0.5 (1 µl : 0.5 µl).

Phylogenetic analysis

To build a phylogenetic tree, the amino acid sequences of 29 functionally characterized tick serpins and one human serpin were retrieved from GenBank or relevant research articles. The selected serpins together with their accession numbers, percentage identities to Iripin-1, and references are listed in **Supplementary Table 4**. The serpin sequences without signal peptides were aligned in Geneious Prime 2022.1.1 (Biomatters) using the aligner MUSCLE 3.8.425 (7). The best amino acid substitution model, the Whelan and Goldman (WAG) + G + I model (20), was chosen with the help of the software MEGA11 (21). The phylogenetic tree was constructed in Geneious Prime 2022.1.1 using the maximum likelihood-based program PhyML 3.3.20180214 (22). The reliability of the tree branching pattern was determined by bootstrapping. Bootstrap values were calculated based on 1,000 replications.

Determination of inhibition constants

The second-order rate constants for the inhibition of trypsin, kallikrein, matriptase, and plasmin by Iripin-1 were measured by a discontinuous method under pseudo-first-order conditions using at least a 10-fold molar excess of the serpin over the protease. Reactions were incubated at room temperature, and incubations were stopped at each time point by the addition of the chromogenic substrate appropriate for the protease used. The slope of the linear increase in color intensity over time gave the residual protease activity at each time point. The apparent (observed) first-order rate constant k_{obs} was calculated from the slope of the plot of the natural log of residual protease activity over time. k_{obs} was measured for six different Iripin-1 concentrations and plotted against the serpin concentration. The slope of this linear plot gave the second-order rate constant k_2. The assay buffer for trypsin, kallikrein, matriptase, and plasmin had the following composition: 20 mM Tris-HCl, 150 mM NaCl, 0.01% Tween 20, pH 7.4. Chromogenic substrates (S-2302 for kallikrein, S-2288 for matriptase and trypsin, and S-2251 for plasmin) were all purchased from DiaPharma Group (West Chester Township, OH, USA) and used at 200 μM final concentration. Final concentrations and origin of proteases used were as follows: 1 nM kallikrein and 50 nM plasmin (Enzyme Research Laboratories, South Bend, IN, USA), 2 nM matriptase (R&D Systems, Minneapolis, MN, USA), and 5 nM trypsin (Sigma-Aldrich, St. Louis, MO, USA).

Expression of cell adhesion molecules in HUVECs (RT-qPCR)

Total RNA was isolated from HUVECs with the help of the NucleoSpin RNA kit (Macherey-Nagel, Düren, Germany) according to the instructions of the manufacturer. Obtained RNA (650–700 ng) was reverse transcribed into cDNA using the Transcriptor First Strand cDNA Synthesis Kit (Roche Diagnostics, Mannheim, Germany). For priming, we utilized an anchored-oligo$(dT)_{18}$ primer only. Five-time diluted cDNA was subsequently used as a template in a PCR reaction that consisted of FastStart Universal SYBR Green Master (Roche Diagnostics), RT-PCR grade water, and gene-specific primers. qPCR was performed in the CFX384 Touch thermal cycler using the CFX Maestro 1.1 software (Bio-Rad Laboratories, Hercules, CA, USA). Cycling conditions were 95 °C for 10 min followed by 42 cycles of 95 °C for 30 sec, 56 °C for 10 sec, and 72 °C for 30 sec. The relative expression of intercellular adhesion molecule-1 (*ICAM1*) and vascular cell adhesion molecule-1 (*VCAM1*) in HUVECs was calculated utilizing the Pfaffl method (23) and two reference genes, glyceraldehyde-3-phosphate dehydrogenase (*GAPDH*) and hypoxanthine phosphoribosyltransferase 1 (*HPRT1*). *GAPDH* and *HPRT1* were selected from a set of six candidate reference genes with the help of the programs NormFinder (24) and geNorm (25). The nucleotide sequences of used primers and amplicon lengths are provided in **Supplementary Table 1**.

Expression of cell adhesion molecules on the surface of HUVECs (flow cytometry)

HUVECs were detached from a cell culture plate using Accutase (Invitrogen) and then were transferred to FACS buffer (PBS, 0.1% BSA, 0.1% (w/v) sodium azide). Cells in FACS buffer were incubated for 30 min at 4 °C with the following fluorophore-conjugated monoclonal antibodies: anti-CD54 (ICAM-1)-Brilliant Violet 605 (clone HA58) and anti-CD321 (JAM-1)-Brilliant Violet 786 (clone M.Ab.F11) from BD Biosciences (Franklin Lakes, NJ, USA), anti-CD106 (VCAM-1)-PE-Cyanine7 (clone STA) and anti-CD99-APC (clone 3B2/TA8) from Invitrogen, and anti-CD323 (JAM-3)-PE (clone SHM33) from BioLegend (San Diego, CA, USA). After washing with FACS buffer, HUVECs were stained with 7-amino-actinomycin D (7-AAD) solution (Invitrogen) in order to exclude non-viable cells from flow cytometric analysis. Data were collected and analyzed using the NovoCyte 3000 flow cytometer and software NovoExpress 1.4.1 (Agilent Technologies, Santa Clara, CA, USA).

Supplementary Table 1. Nucleotide sequences of primers used in the study.

Gene	Species	Sequence	Amplicon length (bp)
iripin-1	*Ixodes ricinus*	Forward: 5´-GGACAAGACGAACGGAAAGA-3´<br>Reverse: 5´-GGAAATCCGCCACATCAATTT-3´	245
rps4		Forward: 5´-GGTGAAGAAGATTGTCAAGCAGAG-3´<br>Reverse: 5´-TGAAGCCAGCAGGGTAGTG-3´	80
GAPDH	*Homo sapiens*	Forward: 5'-GAAGGTGAAGGTCGGAGTC-3'<br>Reverse: 5'-GAAGATGGTGATGGGATTTC-3'	226
HPRT1		Forward: 5'-CCTGGCGTCGTGATTAGTGAT-3'<br>Reverse: 5'-AGACGTTCAGTCCTGTCCATAA-3'	131
ICAM1		Forward: 5'-CAGTCACCTATGGCAACGACT-3'<br>Reverse: 5'-CTCTGGCTTCGTCAGAATCAC-3'	179
VCAM1		Forward: 5'-AGTTGAAGGATGCGGGAGTAT-3'<br>Reverse: 5'-GGATGCAAAATAGAGCACGAG-3'	143

Supplementary Table 2. Concentrations of enzymes used in the screening of protease inhibition by Iripin-1.

Enzyme	Amount of enzyme used (nM)	Remaining enzymatic activity after addition of 200 nM Iripin-1 (%)
α-chymotrypsin	0.025	67.6 ± 4.2
β-tryptase	0.017	103.7 ± 1.2
cathepsin G	9.9	78.8 ± 1.1
chymase	0.45	97.1 ± 0.4
elastase	0.07	46.4 ± 1.5
factor Xa	0.33	68.7 ± 0.9
factor XIa	0.06	93.8 ± 1.1
factor XIIa	0.1	84 ± 2
kallikrein	0.04	42.1 ± 1.3
matriptase	0.03	42.7 ± 0.5
plasmin	1.6	14.4 ± 1.1
proteinase 3	8.5	102.1 ± 4
thrombin	0.01	100 ± 1.9
tissue plasminogen activator (tPA)	0.018	68 ± 1.9
trypsin	0.1	28.3 ± 1.9
urokinase plasminogen activator (uPA)	0.25	85 ± 0.3

Supplementary Table 3. Data collection and refinement statistics.

Data collection	
X-ray diffraction source	BL14.2, BESSY II, Germany
Wavelength (Å)	0.9184
Detector	PILATUS 6M
Crystal-detector distance (mm)	346.829
Rotation range per image (°)	0.10
Total rotation range (°)	360.0
Exposure time per image (s)	0.10
Resolution range (Å)	50.00-2.10 (2.33-2.10)
Space group	$P12_11$ (4)
Unit-cell dimensions: a, b, c (Å)	48.82 91.01 95.84
Unit-cell dimensions: α, β, γ (°)	90.00 97.53 90.00
Mosaicity (°)	0.193
Total number of reflections	331018 (52480)
Number of unique reflections	48350 (7680)
Average I/σ(I)	11.12 (1.62)
Completeness (%)	99.1 (97.7)
CC ½	99.8 (68.9)
R_{meas} (%)[a]	116.8 (14.3)
Overall B factor from Wilson plot (Å^2)	31.0
**Refinement**	
Resolution range (Å)	48.44-2.10
Number of reflections in working set	45891
Final R value (%)[b] / Final R_{free} value (%)[c]	0.190/0.251
Mean B value (Å)	38.591
Number of atoms in the asymmetric unit:	
Protein	5669
Ligand-Magnesium ion	1
Water	502
Total	6191
R.m.s. deviations	
Bonds (Å)	0.007
Angles (°)	1.417
Average B factors (Å^2) Overall	41.0
Ramachandran plot:	
Most favored (%)	97.42
Allowed (%)	0.14
Molprobity score	1.37 (99th percentile) *
PDB code	7QTZ

The data in the parentheses refer to the highest-resolution shell.

[a] $R_{meas} = (|I_{hkl} - \langle I \rangle|)/I_{hkl}$, where the average intensity ⟨I⟩ is taken over all symmetry equivalent measurements, and I_{hkl} is the measured intensity for any given reflection.

[b] R value = $||F_o| - |F_c||/|F_o|$, where F_o and F_c are the observed and calculated structure factors, respectively.

[c] R_{free} is equivalent to R value but is calculated for 5% of the reflections chosen at random and omitted from the refinement process.

*(N=11758, 2.10Å ± 0.25Å) 100th percentile is the best among structures of comparable resolution; 0th percentile is the worst.

Supplementary Table 4. Serpins used in the phylogenetic analysis.

Serpin name	Species	GenBank accession number	Percentage identity with Iripin-1	References
A1AT	*Homo sapiens*	AAB59495.1	29.2%	(26)
AamS6	*Amblyomma americanum*	ABS87358.1	34.6%	(27)
AAS19		JAI08902.1	44.8%	(28)
AAS27		JAI08961.1	48.9%	
AAS41[(1)]		JAI08957.1	48.3%	(28)<br>(29)
Dromaserpin[(2)]	*Hyalomma dromedarii*	Not found	37.1%	(30)
HLS1[(2)]	*Haemaphysalis longicornis*	Not found	32.1%	(31)
HLS2		BAD11156.1	35.6%	(32)
HlSerpin-a		QFQ50847.1	50%	(33)
HlSerpin-b		QFQ50848.1	32.1%	
Ipis-1	*Ixodes persulcatus*	BAP59746.1	31%	(34)
Iripin-1	*Ixodes ricinus*	ABI94055.1	-	(35)
Iripin-3		JAA69032.1	55%	(36)
Iripin-4		ABI94057.1	52.7%	(35)
Iripin-5		JAA71155.1	52%	(37)
Iripin-8		ABI94058.1	42.2%	(35)<br>(38)
Iris		CAB55818.2	31%	(39)
IRS-2		ABI94056.2	57.6%	(35)
IxscS-1E1	*Ixodes scapularis*	AID54718.1	56.1%	(40)
RAS-1	*Rhipicephalus appendiculatus*	AAK61375.1	31.3%	(41)
RAS-2		AAK61376.1	28.2%	
RAS-3		AAK61377.1	34.8%	
RAS-4		AAK61378.1	38.5%	
RHS1	*Rhipicephalus haemaphysaloides*	AFX65224.1	38.1%	(42)
RHS2		AFX65225.1	30.7%	
RHS8		QHU78941.1	45.2%	(43)
RmS-3	*Rhipicephalus microplus*	AHC98654.1	34.8%	(3)
RmS-6		AHC98657.1	48.7%	
RmS-15		AHC98666.1	45.7%	
RmS-17		AHC98668.1	48%	

(1) The full-length amino acid sequence of AAS41 was obtained from the cited article (29) since GenBank contains only a partial sequence of this serpin.

(2) The accession numbers of Dromaserpin and HLS1 were not found, and thus the protein sequences of these serpins were derived from the cited articles (30, 31).

Results

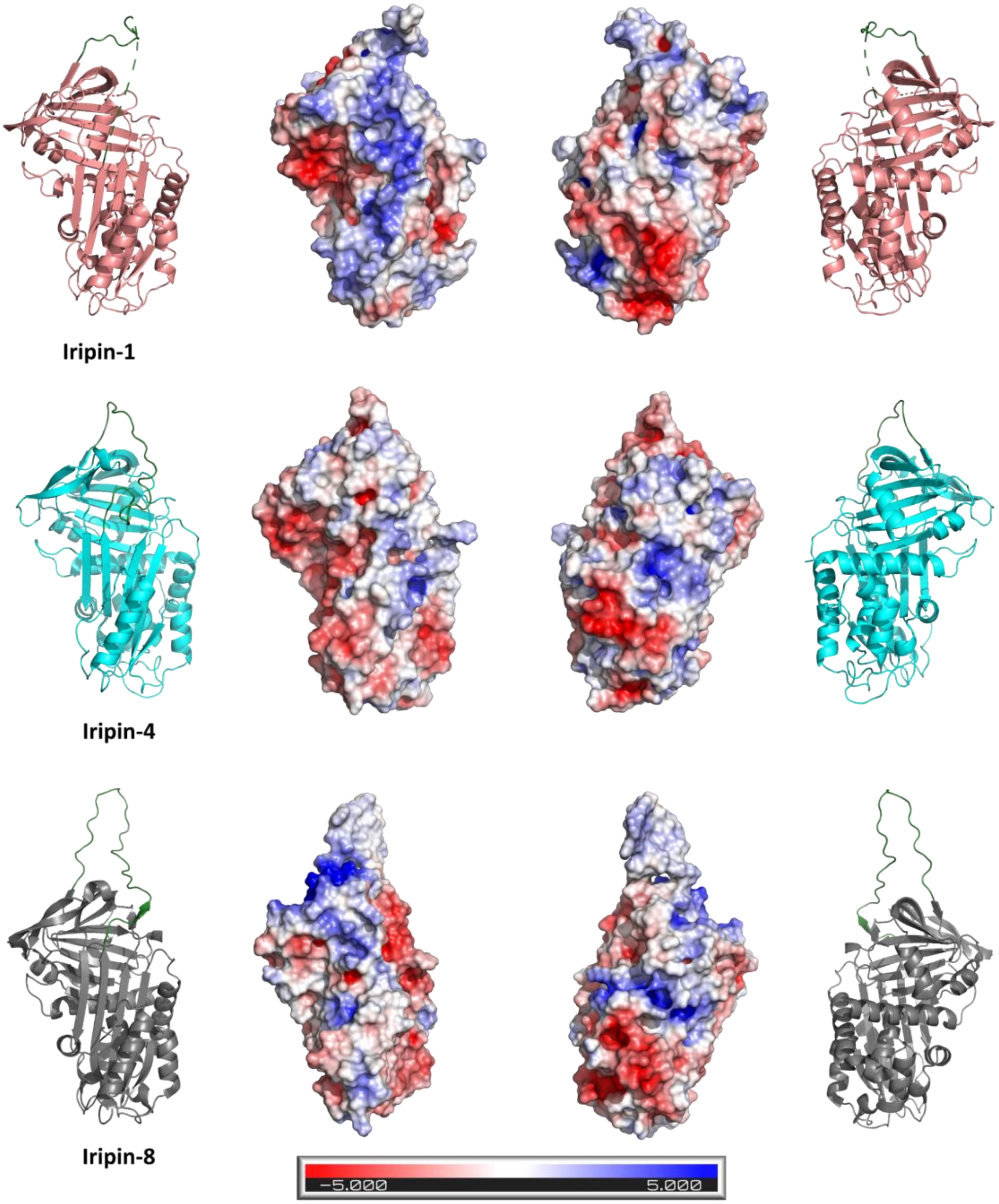

Supplementary Figure 2. Comparison of the surface electrostatic potential of tertiary structures of native *I. ricinus* serpins Iripin-1, Iripin-4, and Iripin-8. The reactive center loops of Iripin-1 (PDB code 7QTZ), Iripin-4 (PDB code 7ZBF) (Kascakova et al., submitted manuscript), and Iripin-8 (PDB code 7PMU) (38) are highlighted in green. The electrostatic potential of individual serpin surfaces was calculated on the crystal structures using the Adaptive Poisson-Boltzmann Solver (APBS) Electrostatics Plugin in the program PyMOL (Schrödinger). Different shades of red and blue color represent negatively and positively charged areas, respectively, as shown in the picture legend (-5 kT/e to 5 kT/e). Neutral areas are colored white. Iripin-1 loop regions with missing amino acid residues, depicted in the cartoon representation of the Iripin-1 structure as dashes, were modelled to display the electrostatic potential map.

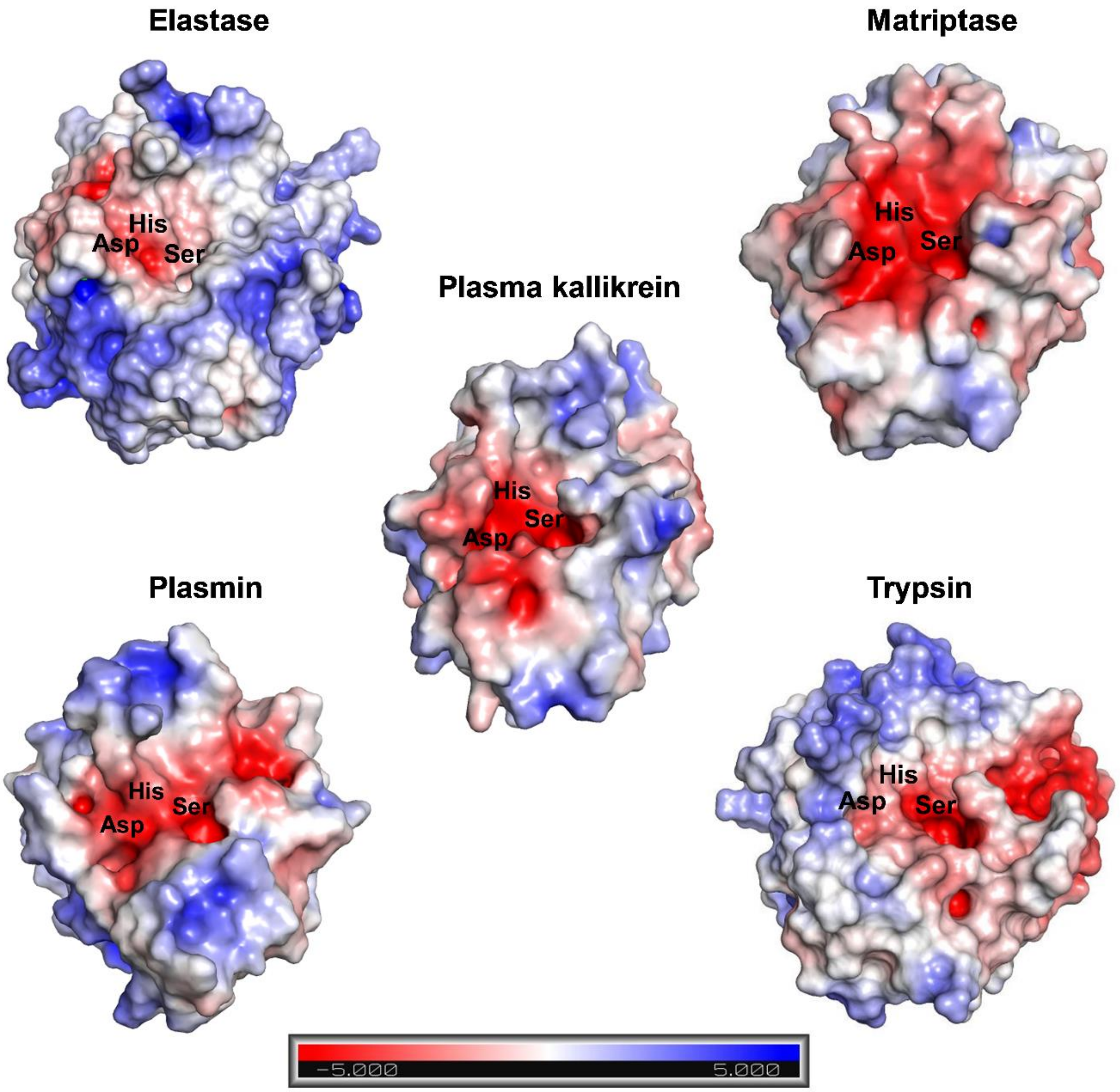

Supplementary Figure 3. Surface electrostatics of active site clefts of selected serine proteases. The surface electrostatic potential of neutrophil elastase (PDB code 3Q76) (44), matriptase (PDB code 1EAX) (45), plasma kallikrein (PDB code 5TJX) (46), plasmin (PDB code 5UGG) (47), and trypsin (PDB code 1TRN) (48) was calculated on the crystal structures using the APBS Electrostatics Plugin in the program PyMOL (Schrödinger). Different shades of red and blue color represent negatively and positively charged areas, respectively, as shown in the picture legend (-5 kT/e to 5 kT/e). Neutral areas are colored white. The aspartate-histidine-serine catalytic triad is labeled in the active site cleft of each protease.

References

1. Gasteiger E, Hoogland C, Gattiker A, Duvaud S, Wilkins MR, Appel RD, et al. *Protein identification and analysis tools on the ExPASy server*. Totowa, New Jersey: Humana Press (2005).

2. Petersen TN, Brunak S, von Heijne G, Nielsen H. SignalP 4.0: discriminating signal peptides from transmembrane regions. *Nat Methods* (2011) 8(10):785-6. Epub 20110929. doi: 10.1038/nmeth.1701.

3. Tirloni L, Seixas A, Mulenga A, Vaz Ida S, Jr., Termignoni C. A family of serine protease inhibitors (serpins) in the cattle tick *Rhipicephalus* (*Boophilus*) *microplus*. *Exp Parasitol* (2014) 137:25-34. Epub 20131211. doi: 10.1016/j.exppara.2013.12.001.

4. de Castro E, Sigrist CJ, Gattiker A, Bulliard V, Langendijk-Genevaux PS, Gasteiger E, et al. ScanProsite: detection of PROSITE signature matches and ProRule-associated functional and structural residues in proteins. *Nucleic Acids Res* (2006) 34(Web Server issue):W362-5. doi: 10.1093/nar/gkl124.

5. Gupta R, Brunak S. Prediction of glycosylation across the human proteome and the correlation to protein function. *Pac Symp Biocomput* (2002):310-22. doi: 10.1142/9789812799623_0029.

6. Altschul SF, Gish W, Miller W, Myers EW, Lipman DJ. Basic local alignment search tool. *J Mol Biol* (1990) 215(3):403-10. doi: 10.1016/S0022-2836(05)80360-2.

7. Edgar RC. MUSCLE: multiple sequence alignment with high accuracy and high throughput. *Nucleic Acids Res* (2004) 32(5):1792-7. Epub 20040319. doi: 10.1093/nar/gkh340.

8. Mueller U, Förster R, Hellmig M, Huschmann FU, Kastner A, Malecki P, et al. The macromolecular crystallography beamlines at BESSY II of the Helmholtz-Zentrum Berlin: Current status and perspectives. *Eur Phys J Plus* (2015) 130(2015):1-10. doi: 10.1140/epjp/i2015-15141-2.

9. Kabsch W. XDS. *Acta Crystallogr D Biol Crystallogr* (2010) 66(Pt 2):125-32. Epub 20100122. doi: 10.1107/S0907444909047337.

10. Sparta KM, Krug M, Heinemann U, Mueller U, Weiss MS. XDSAPP2.0. *J Appl Cryst* (2016) 49(3):1085-92. doi: 10.1107/S1600576716004416.

11. Winn MD, Ballard CC, Cowtan KD, Dodson EJ, Emsley P, Evans PR, et al. Overview of the CCP4 suite and current developments. *Acta Crystallogr D Biol Crystallogr* (2011) 67(Pt 4):235-42. Epub 20110318. doi: 10.1107/S0907444910045749.

12. Evans P. Scaling and assessment of data quality. *Acta Crystallogr D Biol Crystallogr* (2006) 62(Pt 1):72-82. Epub 20051214. doi: 10.1107/S0907444905036693.

13. Vagin A, Teplyakov A. MOLREP: an automated program for molecular replacement. *J Appl Cryst* (1997) 30(6):1022-5. doi: 10.1107/S0021889897006766.

14. Long F, Vagin AA, Young P, Murshudov GN. BALBES: a molecular-replacement pipeline. *Acta Crystallogr D Biol Crystallogr* (2008) 64(Pt 1):125-32. Epub 20071205. doi: 10.1107/S0907444907050172.

15. Zheng B, Matoba Y, Kumagai T, Katagiri C, Hibino T, Sugiyama M. Crystal structure of SCCA1 and insight about the interaction with JNK1. *Biochem Biophys Res Commun* (2009) 380(1):143-7. Epub 20090121. doi: 10.1016/j.bbrc.2009.01.057.

16. Murshudov GN, Skubak P, Lebedev AA, Pannu NS, Steiner RA, Nicholls RA, et al. REFMAC5 for the refinement of macromolecular crystal structures. *Acta Crystallogr D Biol Crystallogr* (2011) 67(Pt 4):355-67. Epub 20110318. doi: 10.1107/S0907444911001314.

17. Emsley P, Lohkamp B, Scott WG, Cowtan K. Features and development of Coot. *Acta Crystallogr D Biol Crystallogr* (2010) 66(Pt 4):486-501. Epub 20100324. doi: 10.1107/S0907444910007493.

18. Chen VB, Arendall WB, 3rd, Headd JJ, Keedy DA, Immormino RM, Kapral GJ, et al. MolProbity: all-atom structure validation for macromolecular crystallography. *Acta Crystallogr D Biol Crystallogr* (2010) 66(Pt 1):12-21. Epub 20091221. doi: 10.1107/S0907444909042073.

19. Young JY, Westbrook JD, Feng Z, Sala R, Peisach E, Oldfield TJ, et al. OneDep: unified wwPDB system for deposition, biocuration, and validation of macromolecular structures in the PDB archive. *Structure* (2017) 25(3):536-45. Epub 20170209. doi: 10.1016/j.str.2017.01.004.

20. Whelan S, Goldman N. A general empirical model of protein evolution derived from multiple protein families using a maximum-likelihood approach. *Mol Biol Evol* (2001) 18(5):691-9. doi: 10.1093/oxfordjournals.molbev.a003851.

21. Tamura K, Stecher G, Kumar S. MEGA11: molecular evolutionary genetics analysis version 11. *Mol Biol Evol* (2021) 38(7):3022-7. doi: 10.1093/molbev/msab120.

22. Guindon S, Dufayard JF, Lefort V, Anisimova M, Hordijk W, Gascuel O. New algorithms and methods to estimate maximum-likelihood phylogenies: assessing the performance of PhyML 3.0. *Syst Biol* (2010) 59(3):307-21. Epub 20100329. doi: 10.1093/sysbio/syq010.

23. Pfaffl MW. A new mathematical model for relative quantification in real-time RT-PCR. *Nucleic Acids Res* (2001) 29(9):e45. doi: 10.1093/nar/29.9.e45.

24. Andersen CL, Jensen JL, Orntoft TF. Normalization of real-time quantitative reverse transcription-PCR data: a model-based variance estimation approach to identify genes suited for normalization, applied to bladder and colon cancer data sets. *Cancer Res* (2004) 64(15):5245-50. doi: 10.1158/0008-5472.CAN-04-0496.

25. Vandesompele J, De Preter K, Pattyn F, Poppe B, Van Roy N, De Paepe A, et al. Accurate normalization of real-time quantitative RT-PCR data by geometric averaging of multiple internal control genes. *Genome Biol* (2002) 3(7):RESEARCH0034. Epub 20020618. doi: 10.1186/gb-2002-3-7-research0034.

26. Long GL, Chandra T, Woo SL, Davie EW, Kurachi K. Complete sequence of the cDNA for human alpha 1-antitrypsin and the gene for the S variant. *Biochemistry* (1984) 23(21):4828-37. doi: 10.1021/bi00316a003.

27. Mulenga A, Khumthong R, Blandon MA. Molecular and expression analysis of a family of the *Amblyomma americanum* tick Lospins. *J Exp Biol* (2007) 210(Pt 18):3188-98. doi: 10.1242/jeb.006494.

28. Porter L, Radulovic Z, Kim T, Braz GR, Da Silva Vaz I, Jr., Mulenga A. Bioinformatic analyses of male and female *Amblyomma americanum* tick expressed serine protease inhibitors (serpins). *Ticks Tick Borne Dis* (2015) 6(1):16-30. Epub 20140918. doi: 10.1016/j.ttbdis.2014.08.002.

29. Kim TK, Tirloni L, Berger M, Diedrich JK, Yates JR, 3rd, Termignoni C, et al. *Amblyomma americanum* serpin 41 (AAS41) inhibits inflammation by targeting chymase and chymotrypsin. *Int J Biol Macromol* (2020) 156:1007-21. Epub 20200419. doi: 10.1016/j.ijbiomac.2020.04.088.

30. Aounallah H, Fessel MR, Goldfeder MB, Carvalho E, Bensaoud C, Chudzinski-Tavassi AM, et al. rDromaserpin: a novel anti-hemostatic serpin, from the salivary glands of the hard tick *Hyalomma dromedarii*. *Toxins (Basel)* (2021) 13(12):913. Epub 20211220. doi: 10.3390/toxins13120913.

31. Sugino M, Imamura S, Mulenga A, Nakajima M, Tsuda A, Ohashi K, et al. A serine proteinase inhibitor (serpin) from ixodid tick *Haemaphysalis longicornis*; cloning and preliminary assessment of its suitability as a candidate for a tick vaccine. *Vaccine* (2003) 21(21-22):2844-51. doi: 10.1016/s0264-410x(03)00167-1.

32. Imamura S, da Silva Vaz Junior I, Sugino M, Ohashi K, Onuma M. A serine protease inhibitor (serpin) from *Haemaphysalis longicornis* as an anti-tick vaccine. *Vaccine* (2005) 23(10):1301-11. doi: 10.1016/j.vaccine.2004.08.041.

33. Wang F, Song Z, Chen J, Wu Q, Zhou X, Ni X, et al. The immunosuppressive functions of two novel tick serpins, HlSerpin-a and HlSerpin-b, from *Haemaphysalis longicornis*. *Immunology* (2020) 159(1):109-20. Epub 20191110. doi: 10.1111/imm.13130.

34. Toyomane K, Konnai S, Niwa A, Githaka N, Isezaki M, Yamada S, et al. Identification and the preliminary *in vitro* characterization of IRIS homologue from salivary glands of *Ixodes persulcatus* Schulze. *Ticks Tick Borne Dis* (2016) 7(1):119-25. Epub 20150924. doi: 10.1016/j.ttbdis.2015.09.006.

35. Chmelar J, Oliveira CJ, Rezacova P, Francischetti IM, Kovarova Z, Pejler G, et al. A tick salivary protein targets cathepsin G and chymase and inhibits host inflammation and platelet aggregation. *Blood* (2011) 117(2):736-44. Epub 20101012. doi: 10.1182/blood-2010-06-293241.

36. Chlastakova A, Kotal J, Berankova Z, Kascakova B, Martins LA, Langhansova H, et al. Iripin-3, a new salivary protein isolated from *Ixodes ricinus* ticks, displays immunomodulatory and anti-hemostatic properties *in vitro*. *Front Immunol* (2021) 12:626200. Epub 20210301. doi: 10.3389/fimmu.2021.626200.

37. Kascakova B, Kotal J, Martins LA, Berankova Z, Langhansova H, Calvo E, et al. Structural and biochemical characterization of the novel serpin Iripin-5 from *Ixodes ricinus*. *Acta Crystallogr D Struct Biol* (2021) 77(Pt 9):1183-96. Epub 20210823. doi: 10.1107/S2059798321007920.

38. Kotal J, Polderdijk SGI, Langhansova H, Ederova M, Martins LA, Berankova Z, et al. *Ixodes ricinus* salivary serpin Iripin-8 inhibits the intrinsic pathway of coagulation and complement. *Int J Mol Sci* (2021) 22(17):9480. Epub 20210831. doi: 10.3390/ijms22179480.

39. Leboulle G, Crippa M, Decrem Y, Mejri N, Brossard M, Bollen A, et al. Characterization of a novel salivary immunosuppressive protein from *Ixodes ricinus* ticks. *J Biol Chem* (2002) 277(12):10083-9. Epub 20020115. doi: 10.1074/jbc.M111391200.

40. Ibelli AM, Kim TK, Hill CC, Lewis LA, Bakshi M, Miller S, et al. A blood meal-induced *Ixodes scapularis* tick saliva serpin inhibits trypsin and thrombin, and interferes with platelet aggregation and blood clotting. *Int J Parasitol* (2014) 44(6):369-79. Epub 20140228. doi: 10.1016/j.ijpara.2014.01.010.

41. Mulenga A, Tsuda A, Onuma M, Sugimoto C. Four serine proteinase inhibitors (serpin) from the brown ear tick, *Rhiphicephalus appendiculatus*; cDNA cloning and preliminary characterization. *Insect Biochem Mol Biol* (2003) 33(2):267-76. doi: 10.1016/s0965-1748(02)00240-0.

42. Yu Y, Cao J, Zhou Y, Zhang H, Zhou J. Isolation and characterization of two novel serpins from the tick *Rhipicephalus haemaphysaloides*. *Ticks Tick Borne Dis* (2013) 4(4):297-303. Epub 20130417. doi: 10.1016/j.ttbdis.2013.02.001.

43. Xu Z, Yan Y, Zhang H, Cao J, Zhou Y, Xu Q, et al. A serpin from the tick *Rhipicephalus haemaphysaloides*: Involvement in vitellogenesis. *Vet Parasitol* (2020) 279:109064. Epub 20200227. doi: 10.1016/j.vetpar.2020.109064.

44. Hansen G, Gielen-Haertwig H, Reinemer P, Schomburg D, Harrenga A, Niefind K. Unexpected active-site flexibility in the structure of human neutrophil elastase in complex with a new dihydropyrimidone inhibitor. *J Mol Biol* (2011) 409(5):681-91. Epub 20110427. doi: 10.1016/j.jmb.2011.04.047.

45. Friedrich R, Fuentes-Prior P, Ong E, Coombs G, Hunter M, Oehler R, et al. Catalytic domain structures of MT-SP1/matriptase, a matrix-degrading transmembrane serine proteinase. *J Biol Chem* (2002) 277(3):2160-8. Epub 20011105. doi: 10.1074/jbc.M109830200.

46. Li Z, Partridge J, Silva-Garcia A, Rademacher P, Betz A, Xu Q, et al. Structure-guided design of novel, potent, and selective macrocyclic plasma kallikrein inhibitors. *ACS Med Chem Lett* (2017) 8(2):185-90. Epub 20161206. doi: 10.1021/acsmedchemlett.6b00384.

47. Law RHP, Wu G, Leung EWW, Hidaka K, Quek AJ, Caradoc-Davies TT, et al. X-ray crystal structure of plasmin with tranexamic acid-derived active site inhibitors. *Blood Adv* (2017) 1(12):766-71. Epub 20170509. doi: 10.1182/bloodadvances.2016004150.

48. Gaboriaud C, Serre L, Guy-Crotte O, Forest E, Fontecilla-Camps JC. Crystal structure of human trypsin 1: unexpected phosphorylation of Tyr151. *J Mol Biol* (1996) 259(5):995-1010. doi: 10.1006/jmbi.1996.0376.
